# Distinct roles of Constitutive Photomorphogenesis Protein 1 homolog (COP1) in human hepatocyte models

**DOI:** 10.3389/fmolb.2025.1548582

**Published:** 2025-02-07

**Authors:** Sébastien Soubeyrand, Paulina Lau, Ruth McPherson

**Affiliations:** ^1^ Atherogenomics Laboratory, University of Ottawa Heart Institute, Ottawa, Canada; ^2^ Department of Medicine, Ruddy Canadian Cardiovascular Genetics Centre, University of Ottawa Heart Institute, Ottawa, Canada

**Keywords:** COP1, HNF4A, hepatocarcinoma, hepatoblastoma, hepatocyte, HepG2, HuH-7, MTTP, TRIB1

## Abstract

**Introduction:**

Constitutive Photomorphogenesis Protein 1 homolog (COP1) is a conserved E3 ligase with key roles in several biological systems. Prior work in hepatocyte-derived tumors categorized COP1 as an oncogene, but its role in untransformed hepatocytes remains largely unexplored. Here, we have investigated the role of COP1 in primary human hepatocytes and two transformed hepatocyte models, HepG2 and HuH-7 cells.

**Methods:**

The role of COP1 was tested by silencing and transduction experiments in HepG2, HuH-7, and primary human hepatocytes. Transcription array data of COP1-suppressed cells were generated and analyzed using clustering analyses. Cellular impacts were examined by proliferation assays, qRT-PCR, western blotting, reporter assays, and APOB enzyme-linked immunosorbent assays.

**Results and Discussion:**

COP1 suppression had no noticeable impact on HepG2 and HuH-7 proliferation and was associated with contrasting rather than congruent transcriptome changes. Transcriptomic changes were consistent with perturbed metabolism in primary hepatocytes and HepG2 cells and impaired cell cycle regulation in HuH-7 cells. In HepG2 and primary hepatocytes but not in HuH-7 cells, COP1 suppression reduced the expression of important hepatic regulators and markers. COP1 downregulation reduced hepatic nuclear factor-4 alpha (HNF4A) abundance and function, as assessed by a lower abundance of key HNF4A targets, reduced APOB secretion, and reporter assays. HNF4A function could be restored by introducing a siRNA-resistant COP1 transgene, whereas HNF4A restoration partially rescued COP1 silencing in HepG2 cells. Our results identify and detail a pivotal regulatory role of COP1 in hepatocytes, in part through HNF4A.

## Introduction

The genome is predicted to encode over 600 RING (really interesting new gene) type E3 ligases that catalyze the last step in substrate ubiquitination (Zheng and Shabek, 2017). Constitutive Photomorphogenic Protein 1 (COP1)/*RFWD2* is an essential, evolutionarily conserved E3 ligase that plays a pivotal regulatory role by promoting ubiquitination and degradation of cognate cargos (Ouyang et al., 2020; Vitari et al., 2011; Bianchi et al., 2003; Migliorini et al., 2011; Qi et al., 2006; Dornan et al., 2004; Choi et al., 2011; Kato et al., 2008). This can be achieved via direct binding (e.g., p53) or may require the presence of additional factors such as the *tribbles* proteins for CEBPA and ACC1 or STK40 for p57^kip2^ (Dornan et al., 2004; Song et al., 2020; Li et al., 2024). Of relevance to liver function, COP1 suppresses lipogenesis and gluconeogenesis in hepatocytes by facilitating ATGL, CEBPA, MLXIPL, and FOXO1 degradation (Kato et al., 2008; Bauer et al., 2015; Ishizuka et al., 2014; Spencer et al., 2020; Ghosh et al., 2016).

In addition, COP1 has also been linked to oncogenesis. Hypomorphic *cop1* mice are susceptible to developing tumors, suggesting a protective role (Migliorini et al., 2011). Similarly, COP1 deficiency in mouse prostate increases cell proliferation and hyperplasia (Vitari et al., 2011). In humans with gastric carcinoma, low COP1 levels are associated with a poor prognosis and have been shown to increase proliferation in a gastric cancer cell line (Sawada et al., 2013). By contrast, COP1 induces acute myeloid leukemia in murine overexpression models (Yoshida et al., 2013). Studies have been inconsistent regarding the role of COP1 function in liver models, with reports of tumor suppressor and oncogenic properties (Migliorini et al., 2011; Song et al., 2020; Lee et al., 2010; Sanchez-Barcelo et al., 2016). Recently, increased COP1 expression was reported in liver tumors and was correlated with reduced survival in liver cancer patients (Cao et al., 2023). HepG2 and HuH-7 cells, two well-characterized liver tumor-derived cell populations, have been reported to undergo profound and rapid growth inhibition following COP1 suppression (Lee et al., 2010). COP1 suppression was shown to elicit growth inhibition and apoptotic regression, and in a nude mouse model, *COP1* siRNA reduced xenograft growth (Lee et al., 2010). By contrast, others reported moderate impacts of altered COP1 levels on HepG2 and HuH-7 proliferation (Cao et al., 2023).

HepG2 and HuH-7 cells were established over 40 years ago. HuH-7 is an established cell line derived from a 57-year-old Japanese hepatoma, whereas the HepG2 cell line was derived from the lobectomy specimen of a 15-year-old Caucasian male (Nakabayashi et al., 1982; Aden et al., 1979). Importantly, while both cell lines have a neoplastic origin, HuH-7 are hepatocarcinoma cells (HCC), whereas HepG2 cells are hepatoblastoma (HBB), despite the latter being often erroneously referred to as hepatocarcinoma (López-Terrada et al., 2009). HBB typically share features reminiscent of fetal liver, are less aggressive, and respond better to chemotherapy than HCC (Ranganathan et al., 2020). As expected for transformed cell lines, HuH-7 and HepG2 genomes are rearranged and mutated (Kasai et al., 2018; Zhou B. et al., 2019; Arzumanian et al., 2021; Zhao et al., 2018). Notwithstanding their transformed phenotypes, they are widely used as surrogate primary hepatocyte models. But this comes at a price, as these cells are known to differ substantially from plated hepatocytes. For instance, they display impaired lipoprotein secretion (Meex et al., 2011; Tsai et al., 2007). The 2 cell lines also differ functionally, including in their dependence on glucose and lipogenic ability (Gunn et al., 2017; Berger et al., 2015). In this work, COP1 is investigated in HepG2, HuH-7 cells, and primary hepatocytes to shed light on its role in pathological and physiological liver settings. In the process, we observed substantial discrepancies with previously published work.

## Materials and methods

### Cell culture

HepG2 and HuH-7 cells were obtained from the American Type Culture Collection and the Japanese Collection of Research Biosources Cell Bank, respectively. Passages p8 to p16 (HepG2) and p20 to p25 (HuH-7) were used. HepG2 and HuH-7 cells were maintained in 5 mM Glucose DMEM (Gibco) supplemented with 10% fetal bovine serum and antibiotic supplements (Gibco). All siRNAs were of Silencer™ Select grade (Ambion) to minimize off-targeting. HepG2 and HuH-7 cells were transfected at 30%–50% confluence in 0.5 mL media in 24 well plates using 10 nM siRNA (final) and 1 µL of RNAiMax. The siRNA transfection mix was prepared in 100 µL Optimem (Gibco). Treatment was continuous for 96 h. Cryopreserved primary hepatocytes (HMCPMS), media, and media supplements were obtained from ThermoFisher Scientific. Two distinct lots, originating from two distinct donors, were used: lot Hu8306 (transcription array analysis of COP1 suppressed hepatocytes; 27 YO, BMI 29.5) and Hu8287 (replication with si33 and si91; 49 YO, BMI 19.6) and were thawed in thawing media and then plated in plating media for 6 h in 12-well plates (1 mL media per well) pre-coated with collagen I (1 vial was seeded in 10 and 14 wells, respectively). Media was changed to culture medium which was replaced every 24 h. Replicates (3) consisting of two treatments each (COP1 silencing oligonucleotides and NT1si) were obtained by staggering transfection by 24 h, i.e. 4, 5, and 6 days after initial plating; 10 nM siRNA and 3 µL of RNAiMax in 100 ul Optimem were used per transfection of cells. Cell extracts were obtained by washing 3 × in PBS and either lysing for RNA (TriPure reagent, Roche) or protein extraction (50 mM Tris-HCl, 1% NP40, 0.15 M NaCl, pH 8) supplemented with phosphatase (PhoStop, Roche) and protease inhibitors (Complete, Roche).

### Expression constructs

COP1 and HNF4A were synthesized *in vitro* by BioBasic (Markham, Ontario) and assembled in the lentiviral PLVX plasmid. COP1 and HNF4A are tagged with C-terminal FLAG epitope and Hemagglutinin tags, respectively. COP1 has been codon optimized to be compatible with gene synthesis. Silent mutations over the siRNA targeted region (sequence in Supplementary Materials) were included to confer resistance to COP1 siRNA. Endogenous HNF4A is generated from two distinct promoters and is alternatively spliced. The variant used here corresponds to NP_000448.3/HNF4α1, the canonical and fully active form, and includes a C-terminal HA tag. Sequences are detailed in the supplemental materials section.

### Viral particle generation and stable cell line generation

Viral particles were generated by co-transfection of 293FT cells with the PLVX, PLVXHNF4A(HA), or PLVXCOP1(FLAG) plasmids along psPAX2 and pMD2.G. Viral particles were harvested during the 16–72 h window post-transfection, concentrated with Lenti-X (Clontech) and stored at −80°C. Titers (Multiplicities of Infections (MOIs)) were first determined on HEK293T cells using 3 μg/mL puromycin over 3 days post-infection. For HepG2 cells, MOIs of 0.2 and 0.6 (“Low” and “High”) of the COP1 virus (or PLVX) or an MOI of 1 of the HNF4A virus (or PLVX) were used to infect 20%–30% confluent cells. Media was changed the next day, and selection with puromycin (3 μg/mL) was initiated for 96 h, with siRNA treatment when appropriate. Uninfected HepG2 controls died within 72 h of puromycin treatment.

### Western blotting

SDS-PAGE Gels (8% or 10%) were transferred to nitrocellulose membranes (Bio-Rad) using a Trans-blot apparatus (2.5 A constant, 6 min). Membranes were briefly stained with Ponceau to ensure even loading, fully destained with 3 × 1 min PBS over 3 min, and blocked in Intercept buffer (LI-COR) for 1 h. Blots were then rinsed once in PBS/0.1% Tween and detected in PBS/0.1% Tween for 16 h at antibody concentrations of 0.5–1 μg/mL. Secondary antibodies were from LI-COR (680 and 800) and were used as 1:20,000 dilutions. Four 30-s PBS rinses were performed between incubations. The acquisition was performed on an Odyssey Imager (LI-COR) with the default settings. All images shown were modified for contrast and intensity only (kappa = 1) and were within the dynamic range of the imager. Antibodies used are listed in Supplementary Materials.

### Cell proliferation assay

The proliferation of cells grown in the presence of siRNA for 96 h was measured by the addition of 1/10 media volume of AlamarBlue (FisherScientific) for 1 h. Fluorescence was measured in a Dynatek (Synergy) fluorometer, using 560 nm excitation and 590 nm emission, using AutoScale. Values from a cell-free well control were included and subtracted from the sample values.

### Luciferase reporter assay

HepG2 cells in 24 well plates were transfected at low density (20%–30%) for 3 days in the presence of 10 nM COP1, HNF4A or NT1 siRNAs. Cells were then washed and transfected for 24 h with 0.3 or 0.1 µg of pGL2MTP containing 5% of renilla internal control (pRL-TK; Promega) (Dai and Hussain, 2012). Cells were then lysed in passive lysis buffer (Promega), and Firefly and Renilla luciferase fluorescence were measured using the Dual Luciferase Assay System on a Glomax luminometer (Promega). Values from untransfected controls were subtracted from the sample values. Firefly activity was normalized to the matching Renilla activity to obtain relative luciferase units, which were then normalized to the NTsi values for each experiment.

### APOB enzyme-linked immunosorbent assay (ELISA)

APOB in the media of si33 and si91 was measured by ELISA (Mabtech). Briefly, media from the last 24 h of siRNA treatment was harvested, centrifuged at 1000 g (2 min) to remove cell debris, and frozen at −80 C until analyzed. Thawed media was diluted 10 X in PBS for assay to fit within the linear range of the APOB standard curve. Substrate (1-Step Ultra TMB-ELISA; ThermoFisher Scientific) was added for 10 min and reactions were stopped with 1M sulfuric acid (final). Absorbance (450 nm) was measured on a Dynatek fluorometer (Synergy). Values were corrected to RNA levels (harvested concurrently) and are expressed relative to the matching control siRNA; similar results were obtained without correction (i.e., using media values only).

### Real-time RNA quantification (RT-qPCR)

Total RNA was extracted from culture plates using Tri-Reagent (Roche) and isolated using Direct-Zol RNA miniprep kits (Zymo Research). RNA (200 ng) was reverse transcribed in a 10 µL reaction using a Roche kit, using a 1:1 mixture of oligo dT and random hexamer primers (Roche). The quantification was performed on a LightCycler 480 (Roche). The target of interest values were expressed relative to the corresponding PPIA values using the ΔΔCt method. Oligonucleotide primers are detailed in the Supplementary Materials section.

### Transcription array analysis

RNA from primary hepatocytes and tumor-derived liver cells (3 biological replicate sets, each consisting of a COP1 silenced and an NT1 control), was isolated 96 h post-silencing and sent to The Centre for Applied Genomics (The Hospital for Sick Children, Toronto, Ontario, Canada) for processing. A HuH-7 sample (COP1si replicate) failed bioanalyzer analysis and was discarded. Biotin-labeled cRNA was hybridized on HuGene-2_0-st arrays (Affymetrix) and results were processed with the Expression Console and analyzed with the Transcriptome Analysis Console from Affymetrix using default settings. False Discovery Rates (FDR) were determined using the Benjamini–Hochberg method. The data are available at the Gene Expression Omnibus (GSE206116).

### Bioinformatic analyses

Mapping of transcriptional consequences of COP1 suppression was performed using the WebGestalt interface (http://www.webgestalt.org/) (Liao et al., 2019). Over-representation analysis (ORA) and Gene Set Enrichment analysis (GSEA) were used. ORA is a binary approach leveraging statistically significant transcripts only whereas GSEA uses an aggregate-score using relative expression values to identify enriched categories. The Affymetrix Gene IDs were mapped via WebGestalt. GSEA was performed using the complete transcriptome fold-change dataset. Nominal changes were used for ORA. The background list was “affy hugene 2.0 stv1”. Only FDR significant hits (q < 0.05) are shown. Impacted transcripts were mapped to the Kyoto Encyclopedia of Genes and Genomes (KEGG), Gene Ontology (GO), and Reactome categories (The Gene Ontology Consortium et al., 2023; Kanehisa and Goto, 2000; Kanehisa et al., 2023; Fabregat et al., 2017). For Ingenuity Pathway Analysis (IPA) core analysis, molecules undergoing fold changes >1.5 (ANOVA p < 0.05) as determined by the Transcriptome Analysis Console (Affymetrix) output were selected. The Ingenuity Knowledge Base (Genes + Endogenous Chemicals) was used as the reference set. High confidence and experimentally observed data were included in the analyses. For IPA Upstream Regulator analysis, known targets of upstream regulators present in the dataset provided were examined, and the direction of the changes observed was compared with the expected direction. The enrichment of impacted targets in the sample list *versus* a database of regulator targets was tested using Fisher’s exact test. Metascape analysis was performed using the nominally significant hits of HepG2, HuH-7, and hepatocytes using the default settings (Zhou Y. et al., 2019). Entrez IDs were used as inputs, and the Human Gene HT 2.0 ST ENTREZ IDs were used as background.

### Statistical analyses

As indicated, statistical significance was tested using (paired) Student’s t-test for two groups or one-way ANOVA for multiple treatment groups.

## Results

### COP1 suppression is well tolerated in HepG2 and HuH-7 hepatoma cells

**FIGURE 1:**
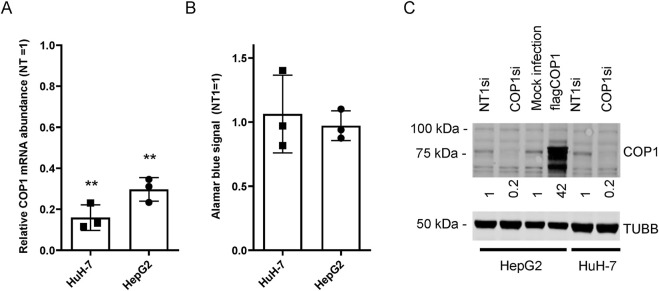
COP1 suppression in HepG2 and HuH-7 cells does not affect proliferation. HuH-7 and HepG2 cells were transfected with a COP1 targeting siRNA (COP1si) or a non-specific control (Non-Target 1; NT1) for 96 h before analysis. **(A)** RNA isolation and quantification by qRT-PCR. **(B)** cell abundance estimation by Alamar blue of siRNA treated cells. Three biological repeats (and means ± SD) are shown. The statistical significance of NT1si vs. COP1si values was assessed using the Students’ t-test. **p < 0.01. **(C)** Western blot of HuH-7 and HepG2 cells treated with siRNA or transduced (for HepG2) with a flagCOP1 expressing virus for 96 h. Quantification representative of three biological repeats. TUBB, Tubulin beta chain.

While examining the role of COP1 in transformed liver models, we found that a 4-day COP1 suppression (∼85% in HuH-7 and ∼70% in HepG2) had little impact on HepG2 and HuH-7 morphology (data not shown) or cell abundance (Figures 1A, B). This observation contrasts with previously published results, showing that a comparable COP1 suppression strongly impaired the proliferation and appearance of both cell types (Lee et al., 2010). Western blotting for COP1 confirmed COP1 suppression at the protein level (Figure 1C). To ensure specificity, HuH-7 and HepG2 cells were treated with two additional siRNAs, neither of which showed toxicity despite comparable COP1 suppression (Supplementary Figure S1). As suppression of COP1 mRNA expression was similar across both studies, other sources of discrepancy were considered, including different transfection reagents, Lipofectamine 2000 *versus* RNAiMAX, used in the current study. We reasoned that Lipofectamine 2000 may synergize with COP1 suppression to be cytotoxic. This was tested in HuH-7; however, the findings were comparable to RNAiMax (Supplementary Figure S2). While the reasons behind this discrepancy were not further investigated, COP1 suppression without significant cell death warranted a fresh assessment of COP1 function in these two cell lines.

### Over-representation analyses of transcriptome changes following COP1 suppression in transformed and primary hepatocytes

HepG2 and HuH-7 transcriptomes following *COP1* silencing were analyzed by transcription arrays (see Supplementary Figure S3 for the volcano plots). Only 423 nominally significant hits were common to HepG2 and HuH-7 cells (7% and 15% of nominal hits, respectively), indicating divergent trajectories with respect to the role of COP1 (Supplementary Figure S3). These hits were mapped to pathways (Reactome and the Kyoto Encyclopedia of Genes and Genomes (KEGG)) and Gene Ontology (GO) terms by over-representation analysis (ORA), which identified enrichment of terms linked to cell cycle regulation and DNA metabolism (Supplementary Table S1). Comparison of nominally impacted transcripts within each cell type revealed dichotomous responses for HepG2 and HuH-7: transcriptome changes in HepG2 mainly mapped to metabolic processes, while in HuH-7, changes were prominently consistent with cell cycle regulation terms (Table 1; Supplementary Table S2).

**TABLE 1:** Over-representation analysis of transcripts nominally affected in COP1 silenced HepG2, HuH-7, and primary hepatocytes. Transcriptome data were mapped to Gene Ontology terms (Biological_Process_noRedundant). The top five FDR significant hits are shown for each cell type. A complete table (including KEGG and Reactome sets) is included as Supplementary Table S2.

	HuH-7	
*Gene Set*	*Description*	FDR
GO:0006260	DNA replication	3.7E-10
GO:0044772	mitotic cell cycle phase transition	4.9E-06
GO:0044843	cell cycle G1/S phase transition	1.1E-05
GO:0000075	cell cycle checkpoint	2.7E-05
GO:0071103	DNA conformation change	2.1E-04

	HepG2	
*Gene Set*	*Description*	FDR
GO:0044282	small molecule catabolic process	4.5E-10
GO:0006631	fatty acid metabolic process	2.2E-06
GO:0006260	DNA replication	1.7E-05
GO:0006732	coenzyme metabolic process	2.2E-04
GO:1901615	organic hydroxy compound metabolic process	4.4E-04

	Hepatocytes	
*Gene Set*	*Description*	FDR
GO:0006631	fatty acid metabolic process	1.1E-06
GO:0044282	small molecule catabolic process	7.8E-06
GO:0015711	organic anion transport	8.0E-06
GO:1901615	organic hydroxy compound metabolic process	8.0E-06
GO:0010876	lipid localization	1.5E-05

**FIGURE 2:**
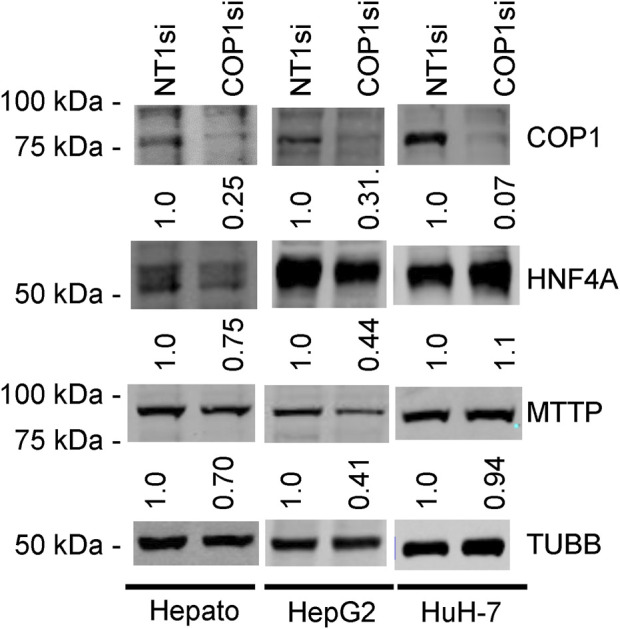
Western blots of COP1si-treated cells. Primary hepatocytes (Hepato), HepG2 cells, and HuH-7 cells were treated with COP1si or a Non-target control (Non-Target 1; NT1) for 96 h. Western blot is representative of two independent biological repeats for primary hepatocytes and four for HepG2 and HuH-7 cells. In each cell type, the same blot was probed sequentially (COP1, MTTP, HNF4A, TUBB). Quantification is shown after correcting for TUBB (Tubulin beta chain) intensity and normalizing to the corresponding NT1si value.

**FIGURE 3:**
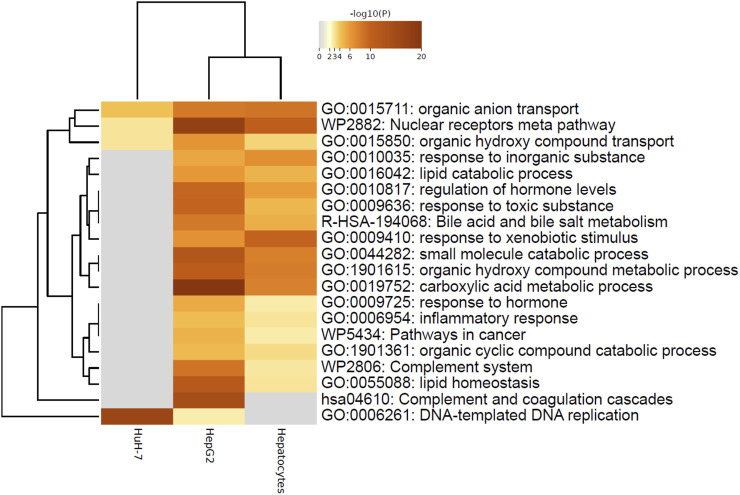
Metascape analysis of nominally significant terms highlighting similarity between HepG2 and Hepatocytes. Analysis was performed on three lists of nominally significant hits (ENTREZ IDs) using Metascape under the default settings. Enriched terms (GO, KEGG, Reactome, Wikipathways) were hierarchically clustered based on Kappa-statistical similarities among their gene memberships. The terms shown represent the lowest p-value in the cluster.

These results pointed to the different roles of COP1 in transformed hepatocyte models. To delineate the physiological role of COP1, COP1 was next targeted in primary human hepatocytes. Consistent with transformed models, siRNA-mediated COP1 knock-down resulted in the suppression of COP1 at the mRNA (reduced to 28% ± 10% of the control value, 95% C.I.) and protein levels (reduced to 25% ± 28% of the control value, 95% C.I.) (Figure 2). The suppression resulted in 4,262 nominally changed transcripts, of which a subset was also impacted in HepG2 (953 of 6057) and HuH-7 cells (404 of 2861) (Supplementary Figure S3). Over-representation analysis indicated that primary hepatocytes underwent transcriptome changes mapping primarily to metabolic processes (Table 1; Supplementary Table S2). Mapping specificity was confirmed by transcript randomization (Supplementary Table S3). Clustering analysis of HepG2, HuH-7, and hepatocytes nominal hits, performed through Metascape, highlighted the greater proximity of HepG2 and hepatocytes *vis à vis* COP1 requirement (Figure 3).

### Gene set enrichment analysis analyses of transcripts in COP1-suppressed transformed and primary hepatocytes

The transcriptome changes were then submitted to Gene Set Enrichment Analysis (GSEA) to complement the ORA findings. GSEA leverages a ranking approach rather than the nominal significance of individual hits to map transcripts of interest to gene sets (Subramanian et al., 2005). Importantly, the analysis provides directionality through a normalized effect size estimate. GSEA identified terms in primary hepatocytes and HepG2 cells centered on impaired metabolic processes, whereas terms related to DNA replication and cell cycling inhibition were identified in HuH-7 (Supplementary Table S4). Uniquely, HuH-7 and HepG2 cells shared terms linked to cellular proliferation (Supplementary Figure S4). However, compared to HuH-7 cells, transcripts mapping to DNA replication and proliferation were globally increased in HepG2 cells (Supplementary Table S4).

### Ingenuity pathway analysis identifies HNF4A dysfunction in primary hepatocytes and HepG2 cells following COP1 knock-down

To further delineate the role of COP1, we performed an Ingenuity Pathway Analysis (IPA). Like GSEA, IPA leverages changes in direction and magnitude to infer functionality but uses its own set of curated pathways (“canonical pathways”) and a different analysis pipeline. Mapping transcriptome changes to canonical pathways following COP1si largely supported earlier analyses (Supplementary Figure S5; Supplementary Table S5). In addition, IPA can infer effects on master regulators (“Upstream Regulators”) in line with transcriptome perturbations. The analysis identified several regulators of interest (Supplementary Table S6). Changes in line with reduced HNF4A activity (“HNF4A″, “HNF4alpha dimer”) and HNF1A/B, two master regulators of liver function, were observed in HepG2 and primary hepatocytes but not in HuH-7 cells. Upon verification of the transcriptome-wide changes in the GSE206116 dataset, *HNF4A* abundance was decreased 2.04-fold in HepG2 (p = 0.0001) and trended towards a reduction (1.28-fold (p = 0.11)) in hepatocytes following *COP1* silencing. By contrast, HNF4A was possibly increased (1.24-fold, p = 0.36) in HuH-7 cells.

### COP1 suppression reduces HNF4A and MTTP function in HepG2 cells and primary hepatocytes

Western blot analyses confirmed that reduced *HNF4A* transcript abundance was accompanied by lower protein abundance in primary hepatocytes and HepG2 but not in HuH-7 cells (Figure 2). *MTTP* is a major regulator of very low-density lipoprotein assembly, and its expression requires HNF4A (Dai and Hussain, 2012; Sheena et al., 2005). Consistently, *COP1* silencing resulted in a statistically significant reduction of *MTTP* mRNA in both HepG2 and primary hepatocytes (3.5-fold and 1.69-fold, respectively, in GSE206116). Again, effects in HuH-7 cells differed with a modest upregulation (1.2-fold). Western blot analysis confirmed that MTTP protein abundance was concordantly reduced in hepatocytes and HepG2 cells but unchanged in HuH-7 cells (Figure 2). Finally, COP1 suppression performed in HepG2 cells reduced the reporter activity of an MTTP promoter construct, previously shown to depend on HNF4A, to a similar extent as HNF4A suppression (Supplementary Figure S6) (Dai and Hussain, 2012).

To ensure specificity, COP1 suppression was repeated in a distinct hepatocyte lot, using the other two siRNAs used earlier (si33 and si91). In addition to *HNF4A* and *MTTP*, *CEBPA,* another major liver transcription factor, reduced in GSE206116 (−1.8-fold, p = 7.8e-5), was also measured. COP1 suppression resulted in a trend toward reduced *HNF4A* expression, as well as reduced *CEBPA* and *MTTP* abundance, replicating the earlier findings (Supplementary Figure S7A). Compromised HNF4A function was ascertained by decreased Apolipoprotein B-100 abundance in the media in silenced cells (Supplementary Figure S7B).

### Limited impact of higher COP1 levels on HepG2 cells

The consequences of increased *COP1* expression were examined next by transducing HepG2 cells with *COP1*; HuH-7, wherein COP1 function seemed to deviate further from hepatocytes, were not examined. Silent mutations and a FLAG tag were introduced to render the transcript siRNA-resistant and to facilitate downstream experiments. *TRIB1*, which was upregulated in the transcriptome analysis and plays a role in sustaining HNF4A function, was also included (Soubeyrand et al., 2016; Soubeyrand et al., 2017). Except for COP1, moderate *COP1* overexpression did not alter transcripts tested, although *TRIB1* and *MTTP* were slightly increased with no effects on protein levels other than that of COP1 (Supplementary Figure S8).

### The introduction of a siRNA-resistant COP1 form partially normalizes transcript abundance

**FIGURE 4:**
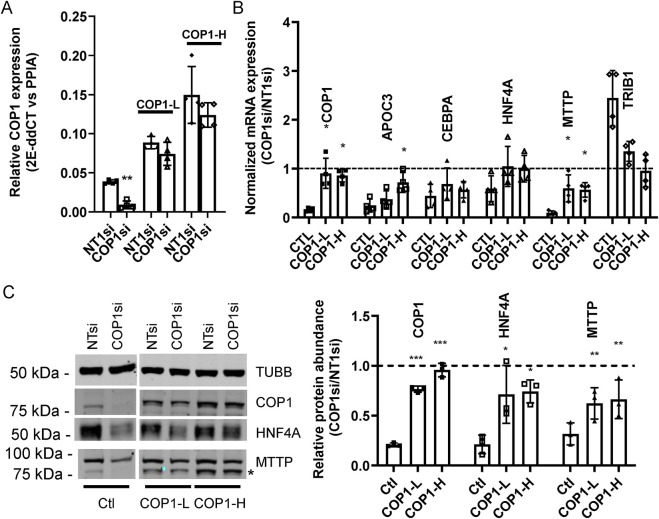
A siRNA-resistant COP1 attenuates the impact of COP1si on hepatic markers. Pools of HepG2 cells, stablytransduced with two doses (COP1-L(ow) and COP1-H(igh)) of a siRNA-resistant form of (flag)COP1 or empty virus (viral dose matching the Wt-H; Ctl) were treated with COP1si or a control siRNA (Non-Target 1; NT1). **(A)** relative expression of COP1 in transduced cells. Levels of COP1 transcript were measured in COP1 or NT1 silenced cells. Statistical significance (NT vs. COP1si) was measured for each transfection using a Student’s paired t-tests. **(B)** qPCR analyses of hepatic markers. The results shown are the COP1si values relative to the corresponding NT1 values. Statistical significance of the COP1 restoration was tested by 1-way repeated ANOVA followed by a *post hoc* Dunnett’s test with the (PLVX) Ctl. Error bars represent standard deviations. **(C)** representative Western blot analysis (left) and quantification of three biological repeats (right). The blot was probed sequentially with COP1 and MTTP; the leftover COP1 signal is indicated by * TUBB, Tubulin beta chain. To test for statistical significance, repeated measures 1-way ANOVAS on the relative (COP1/NT1) protein abundance values (followed by a *post hoc* Dunnett’s comparison test with the control) were performed. Values shown correspond to biological replicates. Bars represent averages ±SD. Errors: *p < 0.05; **p < 0.01; ***p < 0.001. Bars represent the SDC.

Since COP1 overexpression had limited downstream effects, we verified that the siRNA-elicited changes were specific to COP1. COP1 silencing was repeated in pools of HepG2 cells expressing a siRNA-resistant COP1. Introduction of the siRNA-resistant COP1 (resulting in a 2-3-fold increase over basal level) conferred partial protection as assessed by a return towards the baseline of *HNF4A, MTTP, APOC3 (*another prominent target of HFN4A) and*TRIB1* (Figures 4A, B). Changes at the protein level were confirmed by Western blot for HNF4A, MTTP, and COP1, for which suitable antibodies were available (Figure 4C).

### HNF4A silencing in HepG2 cells partially phenocopies COP1 suppression

**FIGURE 5:**
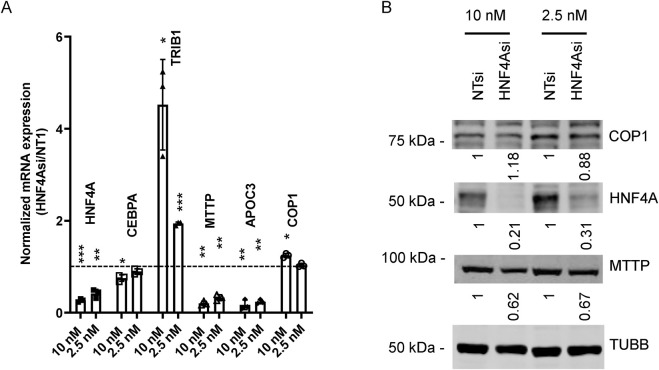
HNF4A suppression partially phenocopies COP1 suppression in HepG2 cells. HepG2 cells were transfected with 10 nM or 2.5 nM siRNA targeting HNF4A or the non-target control (Non-Target 1; NT1) for 96 h before harvest for RNA or protein. **(A)** RT-PCR analysis of transcripts. Values were normalized internally to PPIA levels and are expressed relative to the corresponding NT1 values. Results from three biological replicates (average and SD) are shown. Statistical significance was tested for three experimental repeats against the NT1 value using Student’s t-test. *p < 0.05, **p < 0.01, ***p < 0.001. **(B)** Western blot analysis of corresponding samples. Values were corrected for TUBB (Tubulin beta chain) intensity and normalized to the matching NT1 control. The results shown are representative of three experimental repeats.

COP1 suppression reduced HNF4A protein levels in HepG2 and primary hepatocytes. Since HNF4A is a master regulator of hepatocyte function, we hypothesized that the impacts of COP1 suppression might be through impaired *HNF4A* function (Battle et al., 2006; Hayhurst et al., 2001). First, we confirmed that *HNF4A* suppression via siRNA-mediated silencing phenocopies *COP1* suppression in HepG2 cells (Figure 5A). *HNF4A* suppression reduced *CEBPA*, *MTTP,* and *APOC3* but increased *TRIB1* expression. Interestingly, there was evidence of a dose-dependent relationship. Changes in *HNF4A*, *COP1,* and *MTTP* were further investigated and validated by Western blot (Figure 5B). Unlike *COP1* suppression, which led to diminished *HNF4A* expression, *HNF4A* suppression had a modest impact on *COP1*, suggesting a unidirectional relationship whereby *COP1* regulates *HNF4A* function.

### HNF4A overexpression increases the expression of MTTP and APOC3 in HepG2 cells but does not affect COP1 expression

The consequences of HNF4A overexpression were tested next. The liver-enriched HNF4A form, driven by the P1 promoter (corresponding to NP_000448.3), was transduced in naïve HepG2 cells, and the contribution of increased HFN4A to cognate gene expression was examined. HNF4A overexpression (∼2-fold at the protein level) was sufficient to increase *MTTP* and *APOC3* abundance. By contrast, effects on *CEBPA*, *COP1,* and *TRIB1* were modest, although they reached nominal statistical significance for *CEBPA* and *TRIB1* (Supplementary Figure S9). These findings are consistent with the presence of at least two distinct COP1 regulatory axes in HepG2 cells: a COP1-HNF4A axis regulating *APOC3* and *MTTP* expression and the presence of other COP1-dependent but HNF4A independent (or less dependent) regulatory axes.

### HNF4A overexpression restores MTTP and APOC3 expression but not CEBPA in COP1-silenced HepG2 cells

**FIGURE 6:**
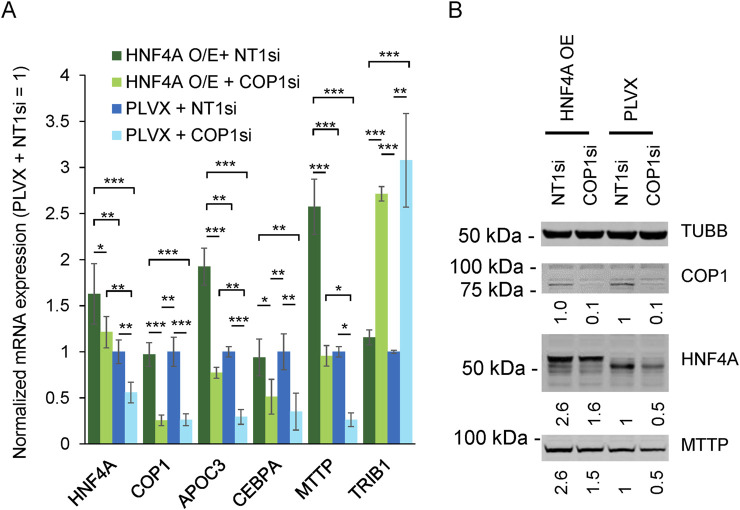
COP1 suppression in HepG2 cells can be partially compensated by HNF4A overexpression. The impact of HFN4A overexpression (O/E) on a selection of targets was assessed by qRT-PCR and Western blotting. **(A)** Transcript abundance assessed qRT-PCR. Values were internally normalized to PPIA expression and are shown relative to the matching mean [PLVX + NT1si] value. Bars represent the means of three independent biological repeats (±S.D.). Statistical significance was tested for each transcript using repeated measures ANOVA followed by Tukey’s *post hoc* tests. Only statistically significant differences are shown. *p < 0.05, **p < 0.01, ***p < 0.001. **(B)** Western blot representative of three biological repeats. Intensities were normalized to TUBB (Tubulin beta chain) and expressed relative to the [PLVX + NT1si] control.

Next, we determined the extent to which impaired HNF4A function contributed to the changes observed following COP1 suppression. The lack of impact of COP1 overexpression on HNF4A protein levels suggested that changes in HNF4A protein abundance in COP1-suppressed cells might stem from reduced *HNF4A* transcription rather than HNF4A degradation. We predicted that the exogenously introduced HNF4A, driven by a heterologous promoter, might resist COP1 silencing and thus allow the contribution of HNF4A under COP1-suppressed conditions to be assessed. To test this hypothesis, *HNF4A* was stably transduced in HepG2 cells. This resulted in a ∼60% increase in *HNF4A* mRNA and a ∼80% increase in HNF4A protein level (Figure 6). Importantly, at comparable *HNF4A* levels (i.e., [HNF4A OE + COP1si] vs. [PLVX + NT1si]), *APOC3* and *MTTP* expression levels were similar, confirming that changes secondary to COP1si can be ascribed to reduced HNF4A abundance. By contrast, HNF4A transduction failed to normalize *CEBPA* and *TRIB1* abundance. Thus, COP1 regulates *TRIB1* and *CEBPA* in part via HNF4A-independent processes.

### Identification of pathways co-regulated by *HNF4A* and *COP1*

To estimate the degree to which processes were co-regulated by HNF4A and COP1, expression profiles of COP1 and HNF4A-suppressed cells were compared. Data from GSE15991, reporting transcriptome changes in *HNF4A*-silenced HepG2, were extracted and compared with our COP1 silenced findings (Wang et al., 2011). Consistent with its pivotal role, *HNF4A* suppression led to the perturbation (i.e., nominally significant changes) of 37% of the HepG2 transcriptome. A comparison of the FDR-significant transcripts affected by HNF4A and COP1 knockdowns revealed a shared set of 215 transcripts out of 4,785 and 515 unique gene symbols for HNF4Asi and COP1si samples, respectively. Over-representation analyses of the overlapping transcripts identified 62 FDR significant Gene Ontology terms, ranging from inflammation to steroid metabolism, and prioritized Reactome and KEGG gene sets linked to bile acid metabolism, peroxisome function, and complement systems (Table 2; Supplementary Table S7). These results suggest that HNF4A and COP1 co-regulate several biological pathways central to liver function.

**TABLE 2:** Over-representation analysis of (FDR) significant transcripts following HNF4A and COP1 silencing. Enriched Gene Ontology terms (Biological_Process_noRedundant) are shown, after simplification by Weighted Set Cover (Webgestalt). A complete table, including KEGG and Reactome sets, is included in Supplementary Tables (Supplementary Table S7).

Gene set	Description	FDR
GO:0044282	small molecule catabolic process	7.4E-07
GO:0015711	organic anion transport	3.5E-05
GO:0070482	response to oxygen levels	2.7E-04
GO:0002526	acute inflammatory response	3.8E-04
GO:0001101	response to acid chemical	4.1E-04
GO:1901615	organic hydroxy compound metabolic process	1.3E-03
GO:0019216	regulation of lipid metabolic process	1.6E-03
GO:0051348	negative regulation of transferase activity	1.7E-03
GO:0043062	extracellular structure organization	2.3E-03
GO:0010038	response to metal ion	5.9E-03

## Discussion

Here, COP1 function was explored in normal and transformed hepatocyte models. A significant discovery was the importance of COP1 in supporting HNF4A function in Hepatocytes and HepG2 cells. More specifically, reduced COP1 abundance inhibited HNF4A-dependent processes. Functional loss was predicted by clustering analyses of microarray data and was ascertained through various experiment approaches, including qRT-PCR quantification and Western blotting, demonstrating reduced HNF4A and MTTP abundance, reporter assays, and diminished APOB secretion in primary hepatocytes.

A comparison of FDR-significant hits in HNF4Asi and COP1si hepatocytes identified unique and shared gene targets, indicating that the functional convergence of HNF4A and COP1 is partial. Transcripts impacted by COP1 silencing represented a small subset (215/4785) of HNF4A-responsive genes, whereas a greater proportion (215/515) of COP1si-impacted genes were also affected by HNF4A silencing, suggesting that the contribution of HNF4A is broader. However, the overlap may be underestimated as several transcripts of potential biological importance were excluded because they did not reach FDR significance. A more permissive approach using nominally significant hits suggests that 20%–25% of the transcriptome requires both COP1 and HNF4A (data not shown). Importantly, as COP1 suppression reduces HNF4A by only 50%–60% (in HepG2s), sufficient HNF4A may persist in sustaining near-normal expression of some HNF4A-dependent genes, further underestimating the actual contribution of COP1 to HNF4A function.

The COP1-HNF4A functional relationship seems largely unidirectional, as COP1 expression was unaffected by HNF4A targeting. Given the pivotal role of HNF4A in hepatocyte function, future work will aim to clarify how COP1 regulates HNF4A levels (Parviz et al., 2003; Santangelo et al., 2011). COP1 is an E3 ligase that can earmark several cognate proteins (CEPBA, MLXIPL, ACC1, FOXO1, ATGL, 14-3-3σ) for degradation (Choi et al., 2011; Ghosh et al., 2016; Hong et al., 2003; Cai et al., 2017). Although HNF4A can be ubiquitylated, our data is inconsistent with a contribution of COP1 (Hou et al., 2024). For one, COP1 suppression led to decreased, rather than increased, HNF4A protein abundance. Moreover, COP1 overexpression did not reduce HNF4A protein level. Thus, the physical interaction we previously reported between HNF4A and TRIB1 is unlikely to contribute to HNF4A degradation; indeed, TRIB1 was positively correlated with HNF4A abundance in that study (Soubeyrand et al., 2017). The contribution of CEBPA, a major regulator of liver homeostasis and a COP1 target, is similarly improbable since it is positively correlated with HNF4A: CEBPA increases HNF4A expression in both cancer models and primary hepatocytes, and CEBPA suppression results in reduced HNF4A (Soubeyrand et al., 2017; Reebye et al., 2018). Consequently, higher CEBPA protein abundance secondary to COP1 suppression should increase rather than reduce HNF4A levels. Indeed, increased CEBPA might dampen the impact of COP1 suppression.

Instead, the breadth of the changes entailed by COP1 suppression is consistent with the implication of multiple effectors. The inability of COP1 overexpression to singly increase HNF4A levels suggests that non-canonical protective functions similar to those identified in *Arabidopsis* are unlikely (Ling et al., 2017). Rather, we propose that COP1 increases *HNF4A* expression by promoting the degradation of HNF4A transcription repressors. An insufficient abundance of obligatory co-regulators may have impeded the impact of COP1 overexpression. Given the ability of COP1 to shuttle across nuclear compartments, cytoplasmic regulators may be targeted (Kung and Jura, 2019). SNAI1 and the NF-kappaB pathway have been shown to inhibit HNF4A transcription (Simó et al., 2012; Cicchini et al., 2006; Zhou et al., 2004). SNAI1 is a transcriptional repressor that antagonizes the hepatocyte-specific program of HNF4A (Cicchini et al., 2006; Garibaldi et al., 2012). SNAI1 is highly unstable and is ubiquitylated by some E3 ligases (Dong and Wu, 2021). With respect to NF-kappaB, GSEA identified increased NF-kappaB signaling in COP1-suppressed HepG2 cells. Furthermore, changes consistent with increased “TNF” (i.e., TNFA) activity were identified by IPA in HepG2 and hepatocytes, consistent with the activation of NF-kappaB signaling in COP1-suppressed cells (Supplementary Table S6). As termination of NF-kappaB signaling via p65/RelA requires proteasome activity, persistent activation from COP1 suppression may inhibit HNF4A expression (Saccani et al., 2004). However, this model is seemingly inconsistent with recent work, based on changed p50/RelA and IκBα abundance, arguing that COP1 promotes NF-kappaB signaling in HepG2 and HuH-7 cells (Cao et al., 2023). Future experiments will investigate these possibilities.

The transcriptional response to COP1 suppression differed significantly in the three cellular models examined. Whereas transcriptome changes elicited by *COP1* suppression clustered to cell regulation and DNA replication terms in HuH-7 and HepG2, perturbations in HepG2 revealed, in addition, a metabolic profile reminiscent of primary hepatocytes. Moreover, *COP1* silencing had dichotomous effects on HepG2 and HuH-7, where transcripts related to cell cycle progression were, as a group, increased in HepG2 but reduced in HuH-7 (Supplementary Figure S10 schematically highlights these differences). This work does not address whether different requirements for COP1 can be attributed to cell line singularities (e.g., specific mutations) or broader features (e.g., hepatoblastoma vs. hepatocarcinoma). For instance, HepG2 (but not HuH-7) has a gain of function mutation in beta-catenin, resulting in constitutive Wnt signaling (de La Coste et al., 1998). However, we note that Wnt signaling (indexed in both Gene Ontology terms and IPA canonical pathways) was not identified by clustering and enrichment analyses of COP1-suppressed cells, despite the role Wnt plays in suppressing some key hepatocyte functions in cancer models, including HepG2 (Nakagawa et al., 2024). Interestingly, IPA upstream analysis predicts a modest inhibition of the related alpha-catenin in HepG2 and primary hepatocytes (Supplementary Table S6). One limitation to these findings is that this 3-way transcriptome-wide comparison hinges on a single siRNA type, albeit designed to minimize off-targeting. While two additional siRNAs had similar impacts on a subset of targets of relevance to hepatocyte function, their transcriptome-wide impacts were not investigated.

Finally, in contrast to previous work, COP1 suppression was well tolerated. Indeed, our transcriptomics findings in HepG2 and HuH-7 differ considerably from those previously published (Lee et al., 2010). In that report, seven transcripts linked to p53 (including RFWD2/COP1) and a list of 78 genes that exhibited directionally coherent changes were reported to be impacted by COP1 silencing. No such pattern was evident from our data (Supplementary Figure S11; Supplementary Table S8). The reasons for this discrepancy are unclear. One possibility is that the previously reported changes resulted from apoptosis engagement, possibly through p53. Indeed, a separate report demonstrated (albeit in non-liver models) that COP1 could mediate p53 turnover, preventing p53-mediated cell death (Dornan et al., 2004). By contrast, reduced COP1 levels were associated with increased incidence of T cell lymphoma in mouse models, suggesting impaired p53 function (Migliorini et al., 2011). Thus, COP1 downregulation combined with other stressors, rather than COP1 suppression *per se*, may sufficiently activate p53 to lead to apoptotic death. Although IPA upstream analysis identified transcriptome-wide changes consistent with p53 activation in HuH-7 cells (but not in HepG2 cells), we did not find evidence that reduced COP1 level is cytotoxic in either cell model, consistent with lack (HepG2) or insufficiency (HuH-7) of p53 engagement (Supplementary Table S6). Whether a more pronounced suppression of COP1 (e.g., via Knock-out) is cytotoxic remains untested in these models. COP1 may not be essential, as COP1-null murine embryos proliferate up to E.9.5 before they die, possibly from cardiovascular malformations (Migliorini et al., 2011). A recent study leveraging COP1-suppressed HepG2 and HuH-7 cells reported a slight reduction in proliferation and DNA replication (Cao et al., 2023). Significantly, suppression was achieved through stable expression of shRNA-expressing vectors, resulting in a greater reduction in COP1 abundance (closer to 90%, although quantification was not provided). In line with our work, shCOP1 and shCTL cell lines showed qualitatively similar proliferation kinetics, demonstrating that a substantial reduction in COP1 abundance is compatible with cell proliferation.

## Data Availability

The datasets presented in this study can be found in online repositories. The names of the repository/repositories and accession number(s) can be found below: Gene Expression Omnibus (https://www.ncbi.nlm.nih.gov/geo/), GSE206116 and GSE15991.

## Supplementary material

SUPPLEMENTARY DATA SHEET S1 Supplementary materials.

SUPPLEMENTARY DATA SHEET S2 Supplementary figures.

SUPPLEMENTARY DATA SHEET S3 Supplementary tables.

## Abbreviations

COP1, Constitutive Photomorphogenesis Protein 1 homolog; HNF4A, hepatic nuclear factor-4 alpha; GSEA, Gene Set Enrichment Analysis; KEGG, Kyoto Encyclopedia of Genes and Genomes; GO, Gene Ontology; ORA, over-representation analysis; IPA, Ingenuity Pathway Analysis.
