# Supplementary material for: Distinct roles of Constitutive Photomorphogenesis Protein 1 homolog (COP1) in human hepatocyte models
Source: Front Mol Biosci. 2025 Feb 7;12:1548582. doi: 10.3389/fmolb.2025.1548582 (PMC11842253; doi:10.3389/fmolb.2025.1548582)
Supplement: Supplementary file 1 [file DataSheet2.pdf]

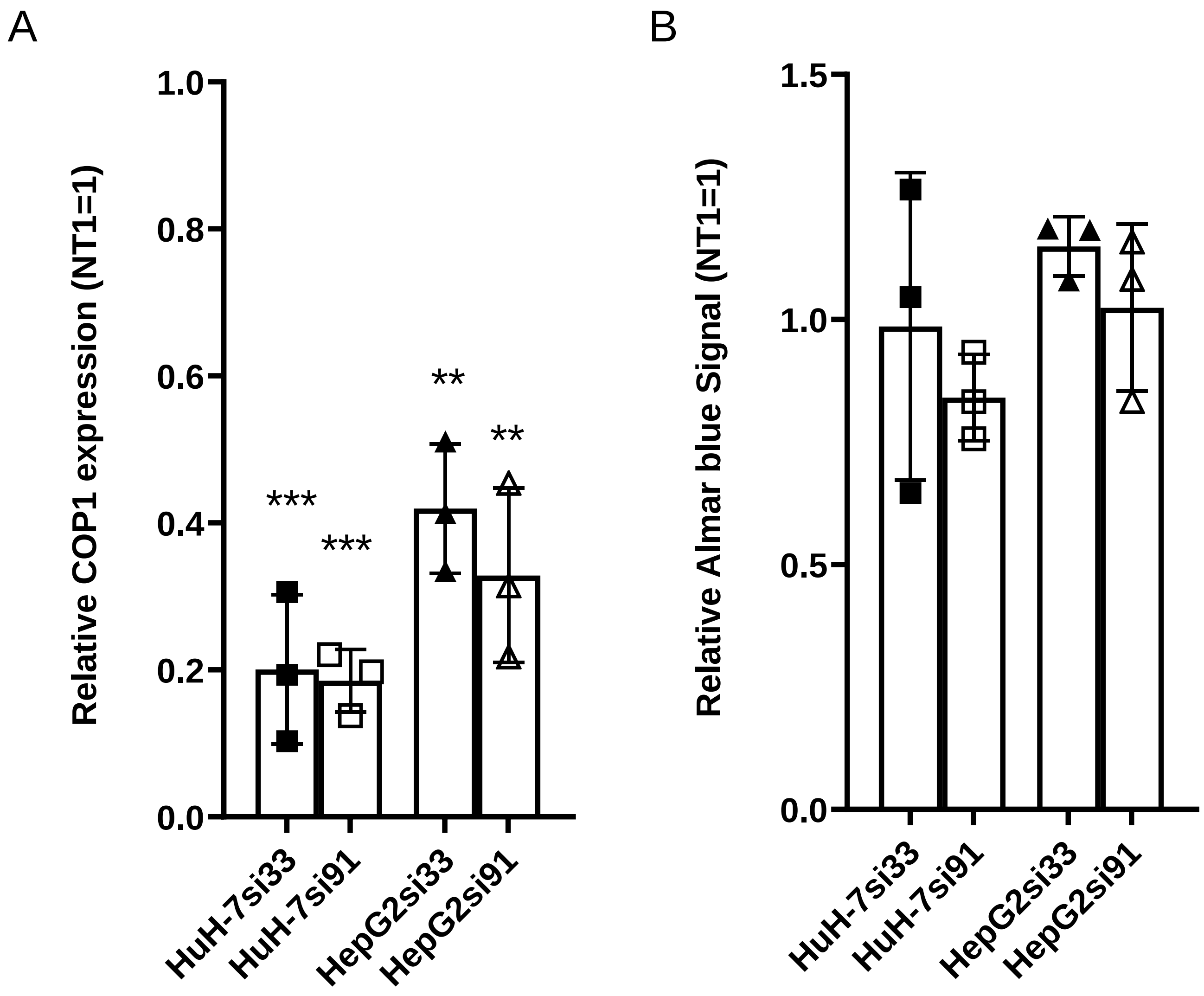

Figure S1. COP1 suppression via distinct siRNA is associated with overtly normal cell growth in cell culture. HepG2 and HuH-7 cells were treated with NT1 (Non-Target 1) siRNA, COP1si-33 or COP1si-91 for 96 h. Cell growth was estimated with Alamar blue (B), while mRNA was quantified by qRT-PCR (A). Results from three biological replicates (average and SD) are shown. Statistical significance of NT1 vs COP1 targeting siRNAs was tested using a Student's t-test. Errors: *, p<0.05; **,p<0.01;***,<0.001.

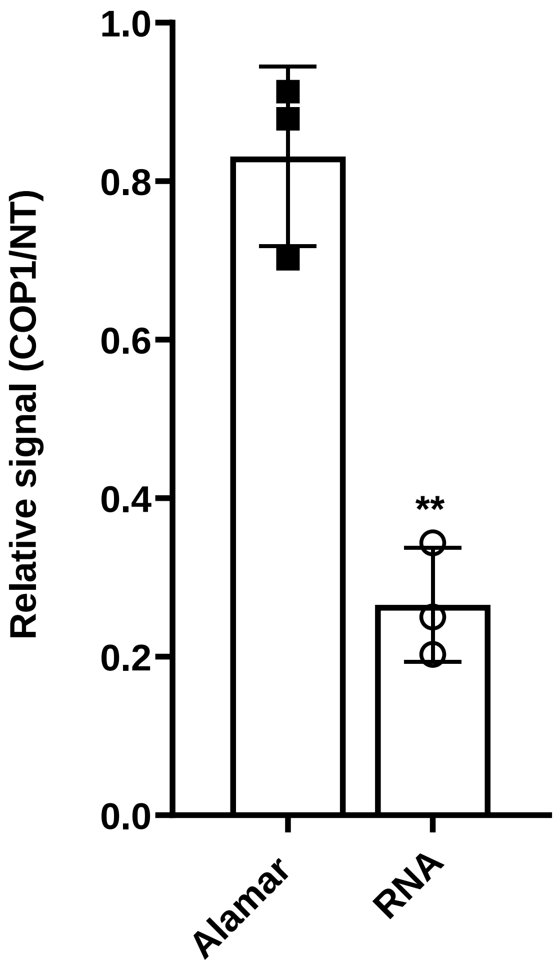

Figure S2. Lipofectamine 2000 mediated COP1 suppression is not toxic in HuH-7 cells. HuH-7 cells were treated for 96 h with Lipofectamine 2000 with either COP1si or Non-Target1 (NT) siRNA. Changes in proliferation (Alamar) and COP1 RNA levels (RNA) were then determined. Alamar signal reduction was not statistically significant (Student's t-test). Results from three biological replicates (average and SD) are shown. **, p<0.001.

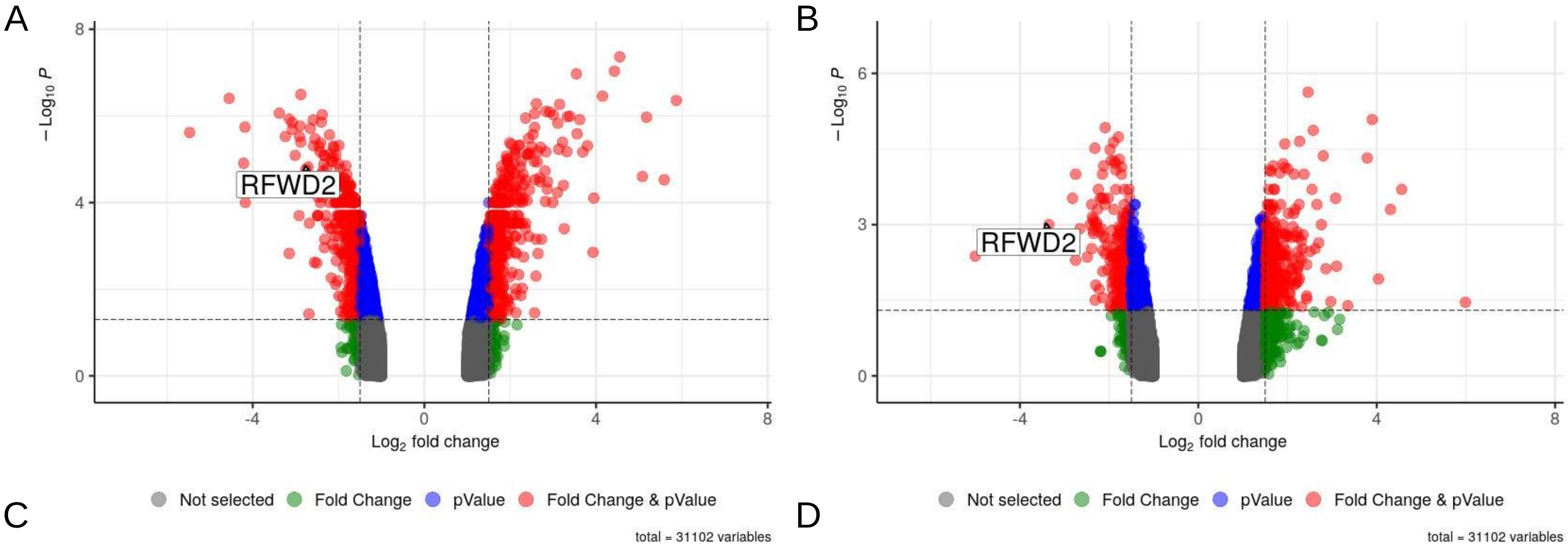

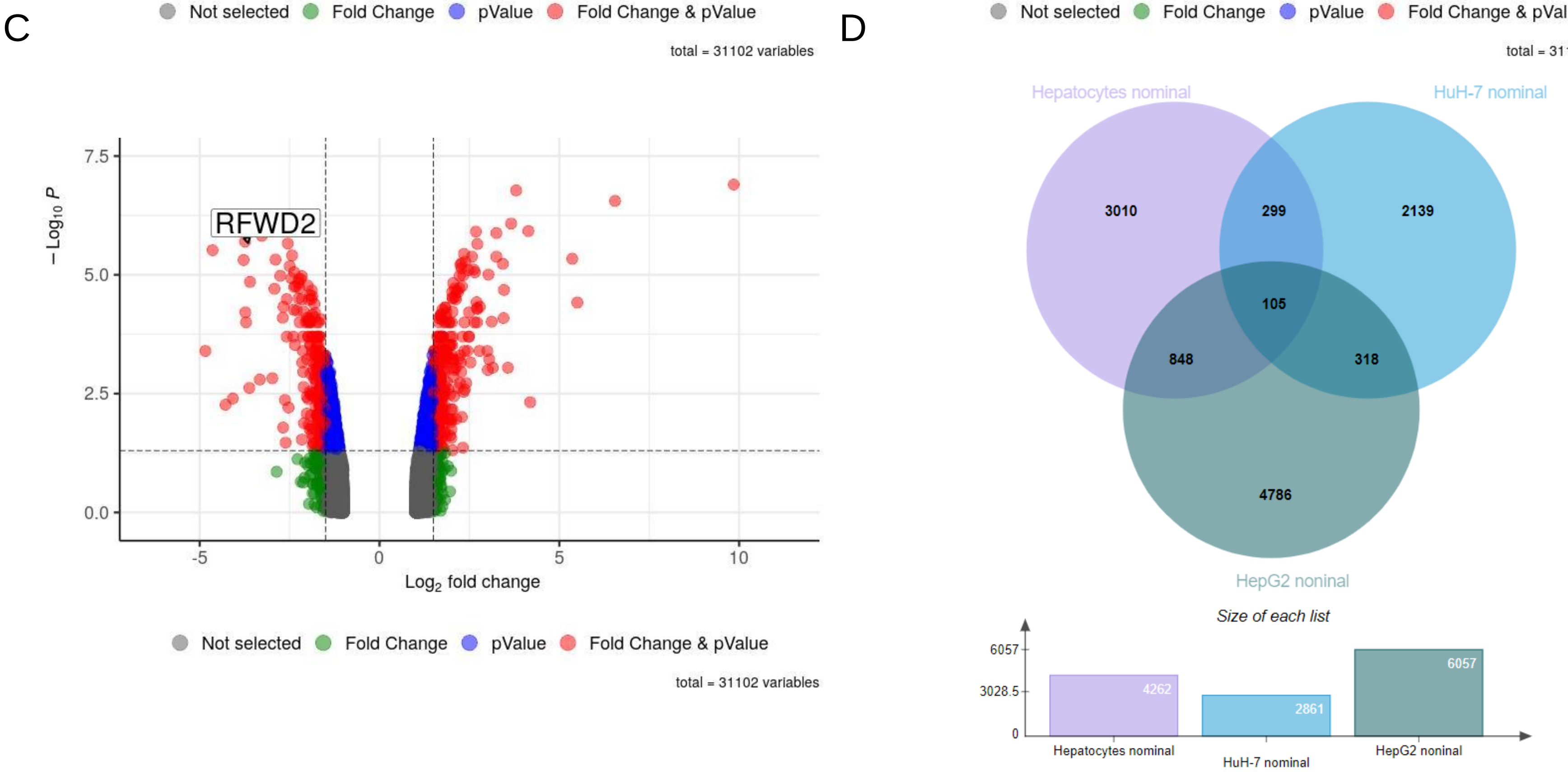

Figure S3. Volcano plots and overlap of significantly changed transcripts in response to COP1 suppression in the three cell models. A-C, Volcano plots of transcript arrays from HepG2, HuH-7 and Hepatocytes. Generated with MaGIC Volcano Plot Tool (https://volcano.bioinformagic.tools). COP1/RFWD2 is highlighted. D, Venn diagram of the overlap between nominally ($p < 0.05$) changed transcripts. Generated with E Venn (http://www.ehbio.com/test/venn/#/).

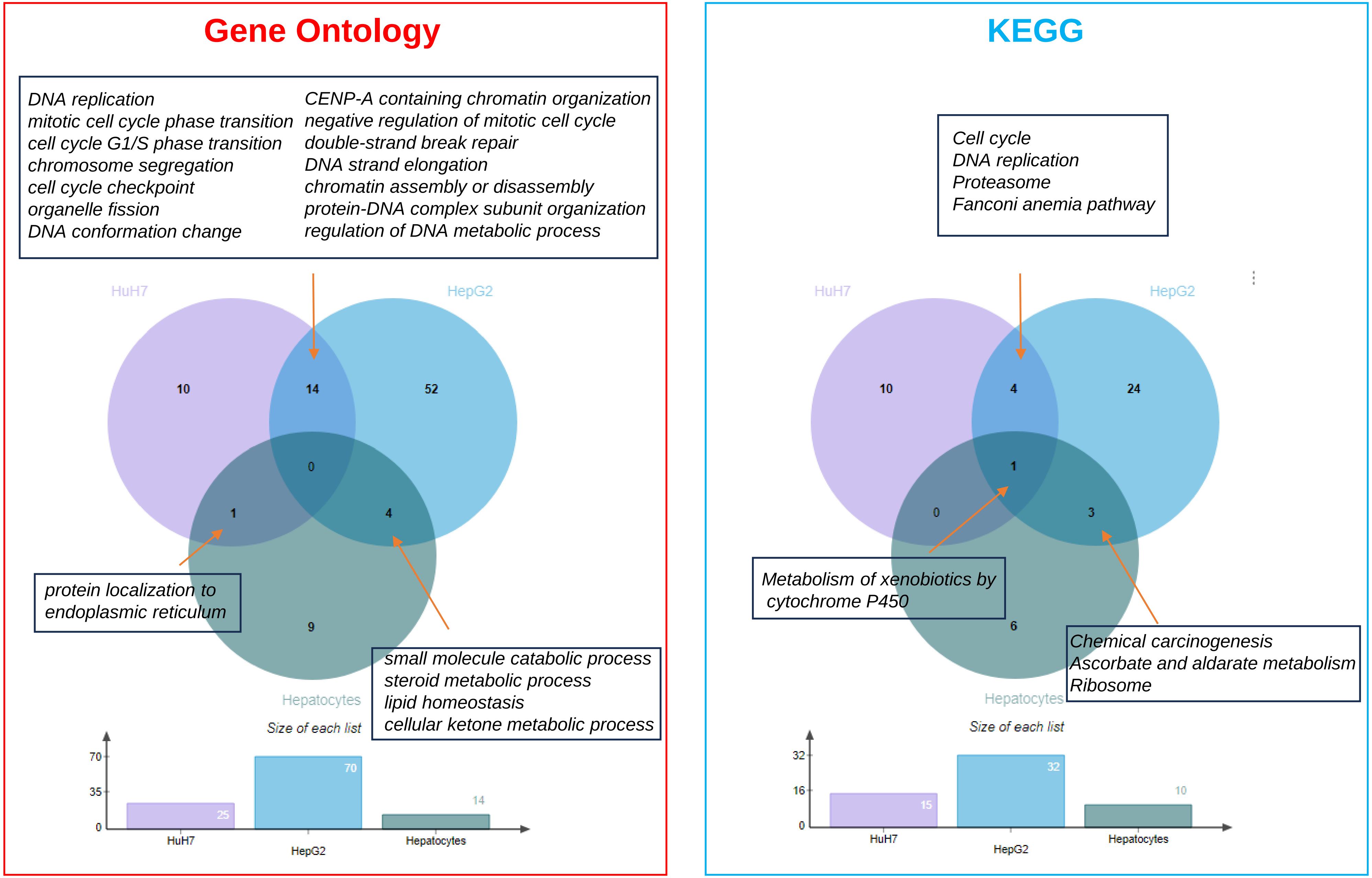

Figure S4. Venn diagrams of GO and KEGG terms enriched in hepatocyte models. Generated in E Venn (//www.ehbio.com/test/venn/#/) using GSEA-identified terms. For KEGG, only FDR <0.01 terms were included to simplify visualization. For the complete list of FDR significant terms, see Table S4.

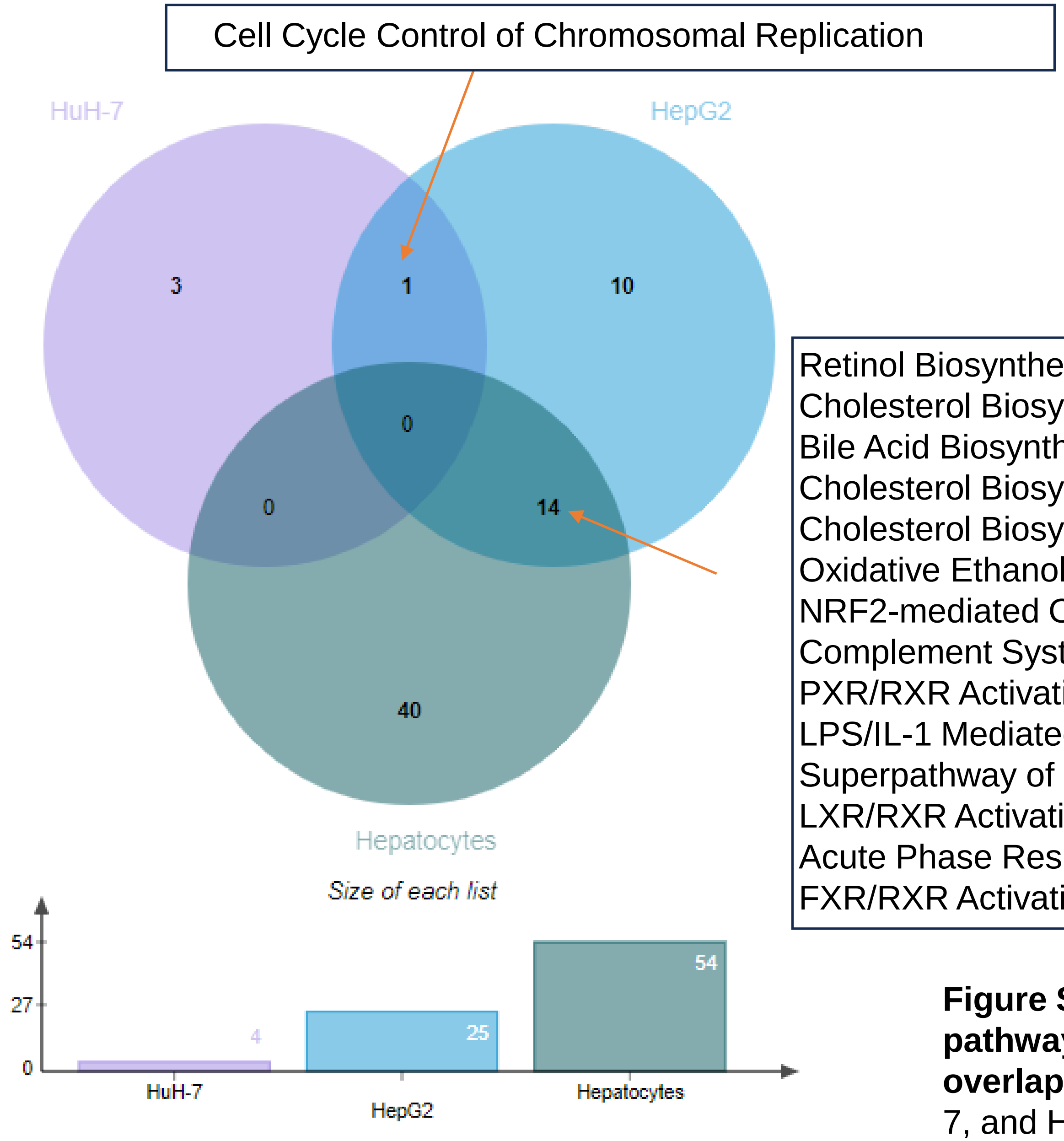

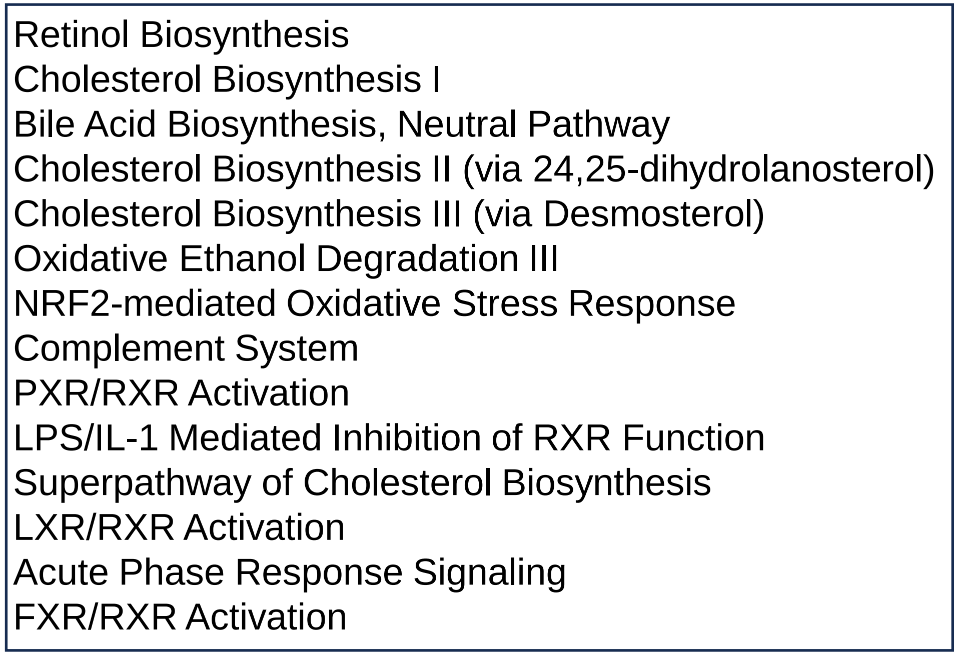

Figure S5. Venn diagram representation of the significant canonical pathways identified by Ingenuity Pathway Analysis, highlighting the overlap between the cell types. Nominally significant hits in HepG2, HuH-7, and Hepatocyte were mapped to ingenuity canonical pathway, yielding a total of 68 B-H significant pathways. Only B-H significant pathways are displayed as a Venn Diagram with E Venn (//www.ehbio.com/test/venn/#/). For the complete list of the 68 pathways identified, see Table S5.

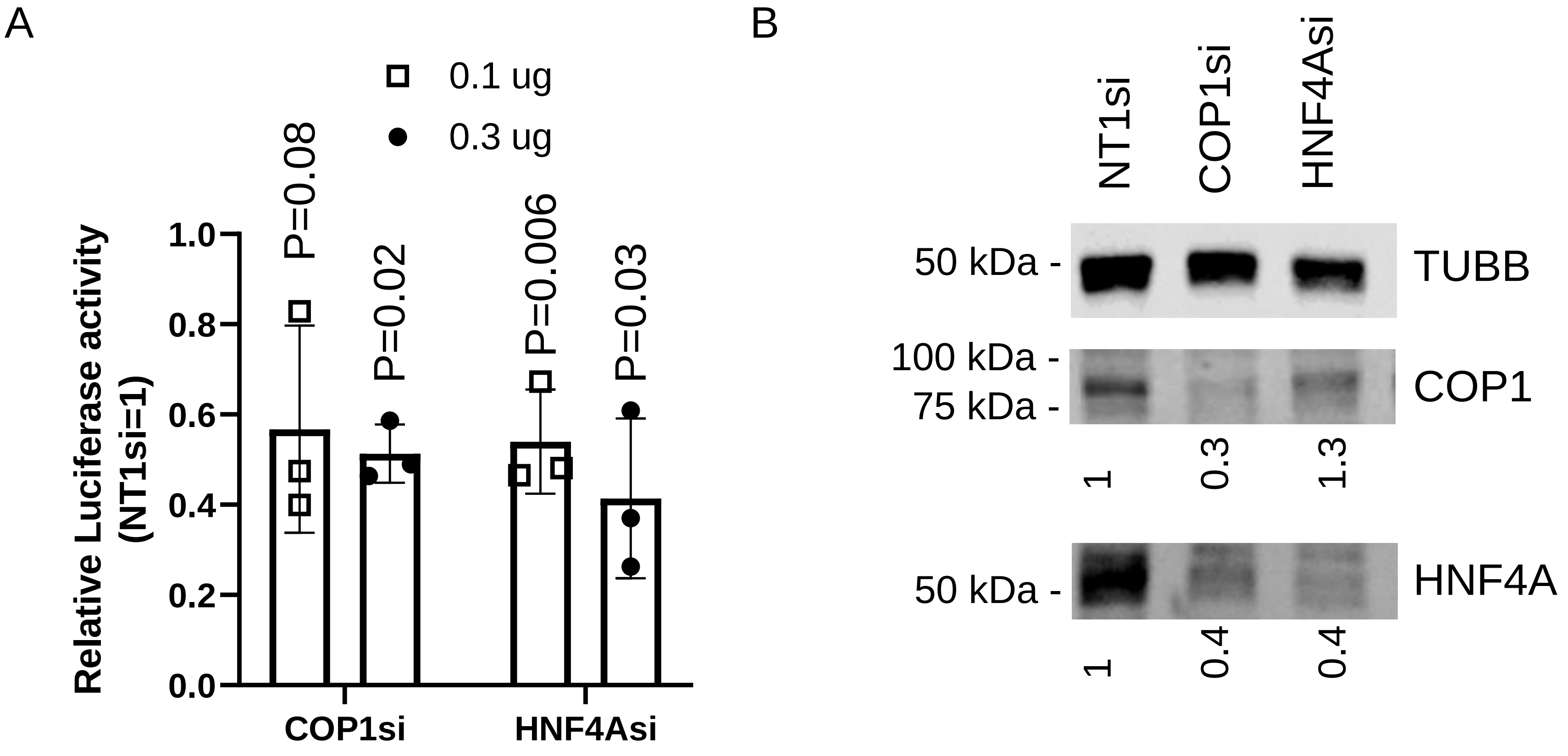

Figure S6. COP1 suppression reduces HNF4A-dependent promoter activity. HepG2 cells were treated for 72 h with either COP1si, HNF4A, or Non-Target1 (NT1) siRNA. Cells were then transfected for 24 h with 0.1 or 0.3 µg of pGL2MTP and pRL-TK to assess HNF4A function. A, Relative fluorescence (Firefly/Renilla) was measured for each sample and the results are expressed relative to the NTsi control. Results from three biological replicates (average and SD) are shown. Statistical significance was determined using a one-sample t-test vs the NT values. B, Western blot of the matching cell lysates, representing 3 biological replicates, with matching quantification. Samples were loaded using a constant lysate volume corresponding to 10-20 ug of protein per lane. Values were normalized to TUBB (Tubulin beta chain) and are expressed relative to the NT1si value.

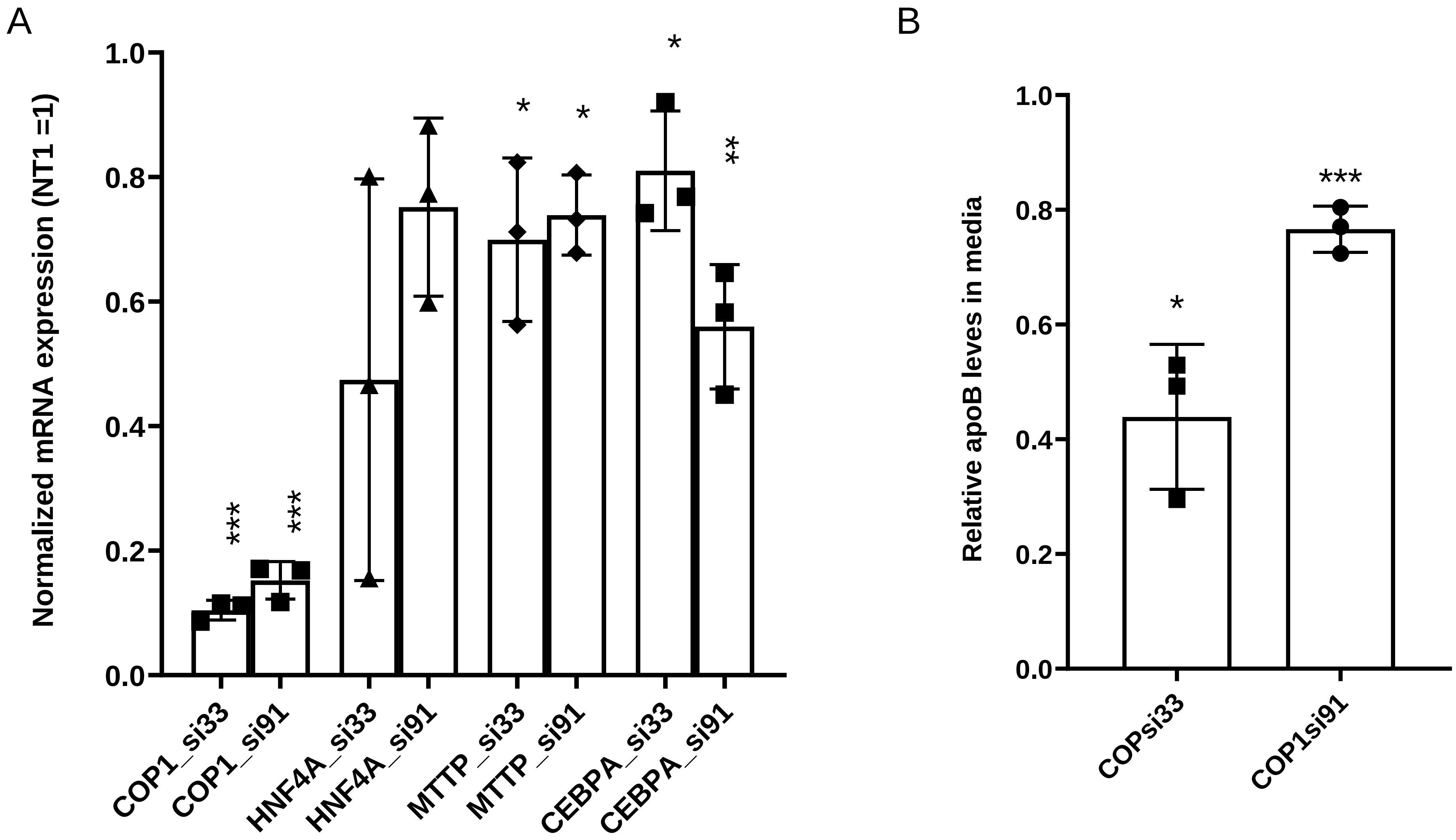

Figure S7. COP1 suppression results in reduced expression of major liver genes and apoB secretion. A, Hepatocytes were transfected with control or two cognate COP1 siRNAs (si33 and si91). RNA abundance was determined by qRT-PCR. Values were normalized internally to PPIA levels and are expressed relative to the corresponding NT1 values. B, Relative apolipoprotein B (apoB) concentration in the media. ApoB concentration was determined by ELISA, divided by the corresponding total RNA concentration (as a proxy of cell number). Values are expressed relative to the matching control (NT1) value. Results from three biological replicates (average and SD) are shown. Statistical significance was determined using a paired t-test vs the NT values. *, $p<0.05$;** $p<0.01$; ***,<0.001.

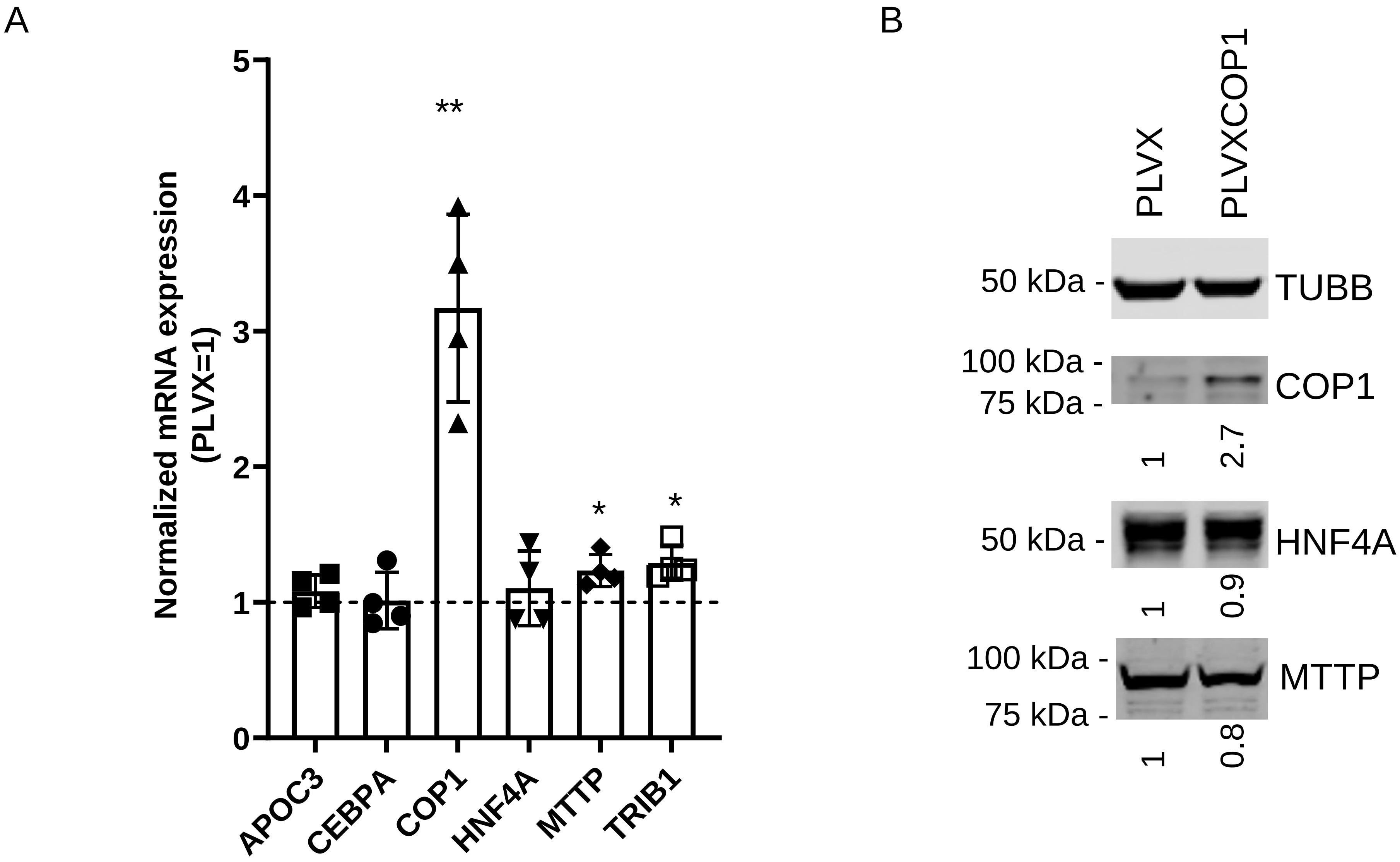

Figure S8. Impact of COP1 overexpression on a panel of key liver transcripts. A, a pool of HepG2 cells, stably transduced with COP1, were examined over four passages for the expression of the indicated transcripts by qRT-PCR. Results are expressed relative to the matching PLVX value. Results from four biological replicates (average and SD) are shown. Statistical significance was measured using the Student's t-test. B, western blot representative of 3 biological repeats. Values were normalized to TUBB (Tubulin beta chain) and are expressed relative to PLVX. Only COP1 protein abundance was significantly affected (3.2-fold, p=0.002, Student's t-test).

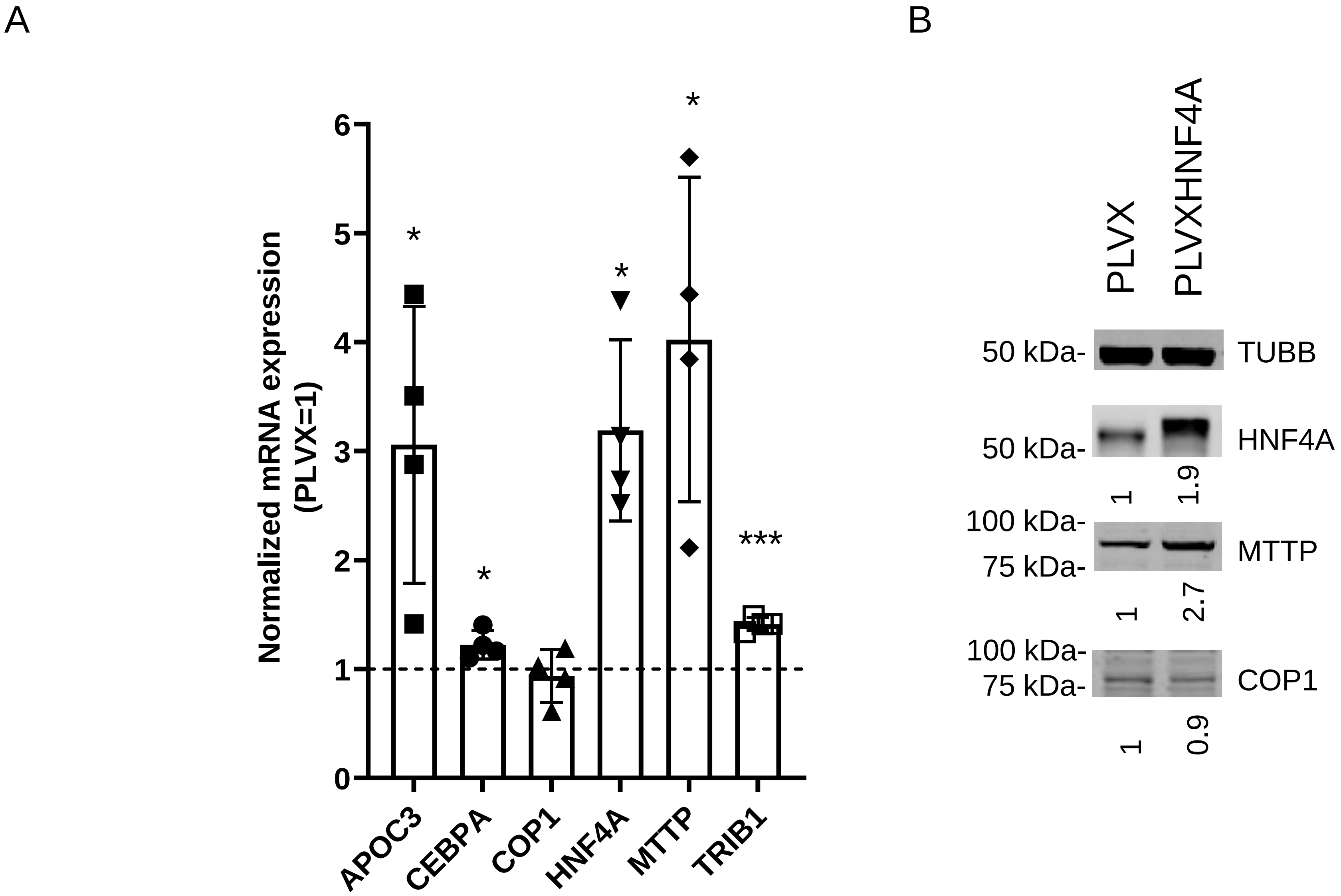

Figure S9. Impact of HNF4A overexpression on a panel of key liver transcripts. Stable pools of HepG2 cells, transduced with viral particles expressing either HNF4A or vector alone, were selected with puromycin and analyzed 10 to 14 days post infection for RNA and protein contents. A, qRT-PCR analyses of transcripts of interest. Values are normalized to the matching control (PLVX only) values. Student's (paired) *t*-test was performed comparing the HNF4 and PLVX values. Results from three biological replicates (average and SD) are shown. *, $p<0.05$;***,<0.001. B, Western blot analysis of protein lysates. Values were normalized to TUBB (Tubulin beta chain) and are expressed relative to PLVX. Representative of 3 experimental repeats.

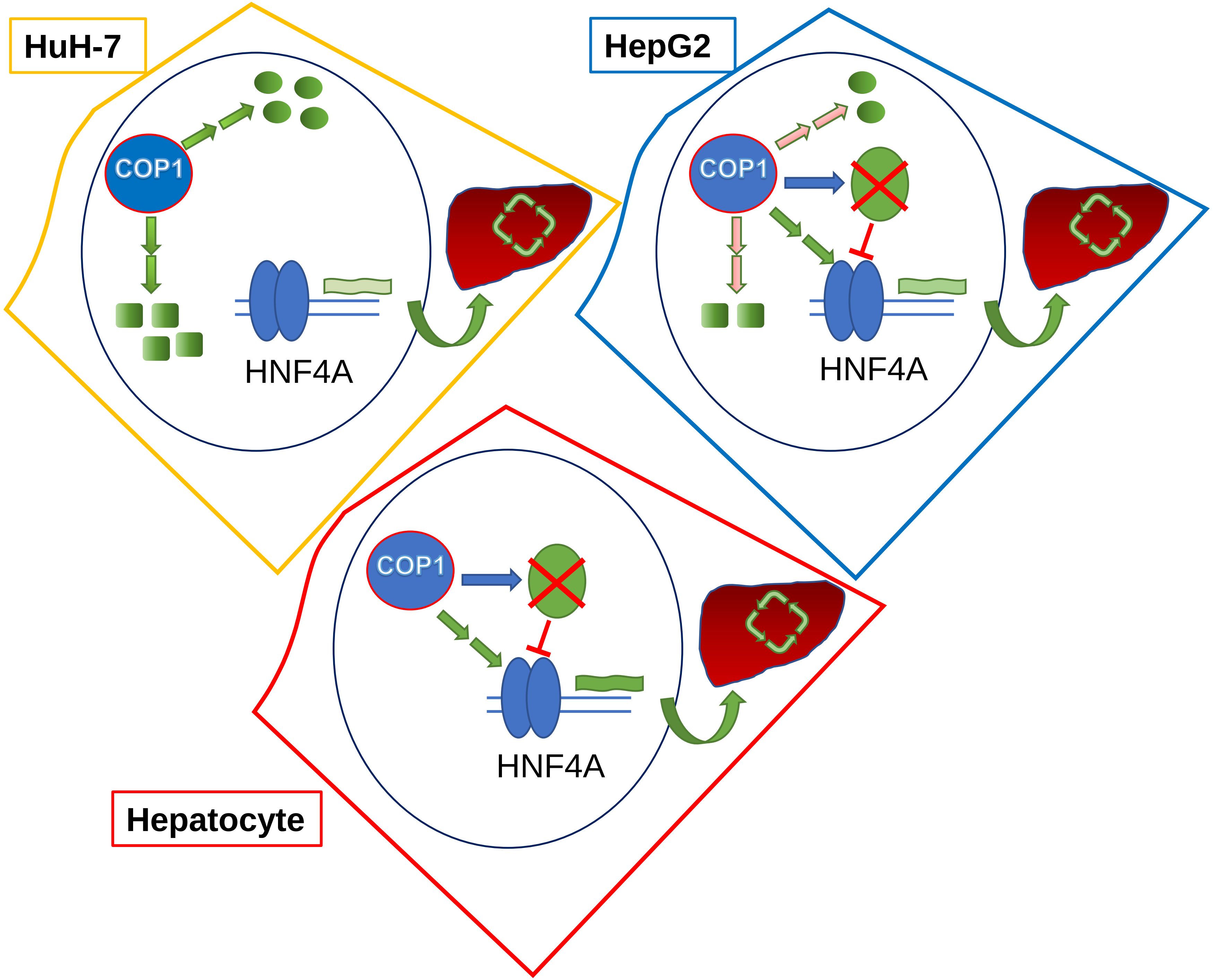

Fig S10. Schema of COP1 function in HuH-7, HepG2 and Hepatocytes. In HuH-7, COP1 and HNF4A are functionally uncoupled. COP1 supports the expression of transcripts associated with cell cycle progression whereas HNF4A is functionally independent, conferring some hepatocyte identity. In HepG2, COP1 contributes to the reduced expression of genes implicated in cell cycle progression. In addition, COP1 sustains HNF4A function by favoring HNF4A abundance, either through interfering with HNF4A degradation or promoting its expression. By contrast, COP1 function in hepatocytes is predominantly metabolic, in part through supporting HNF4A function.

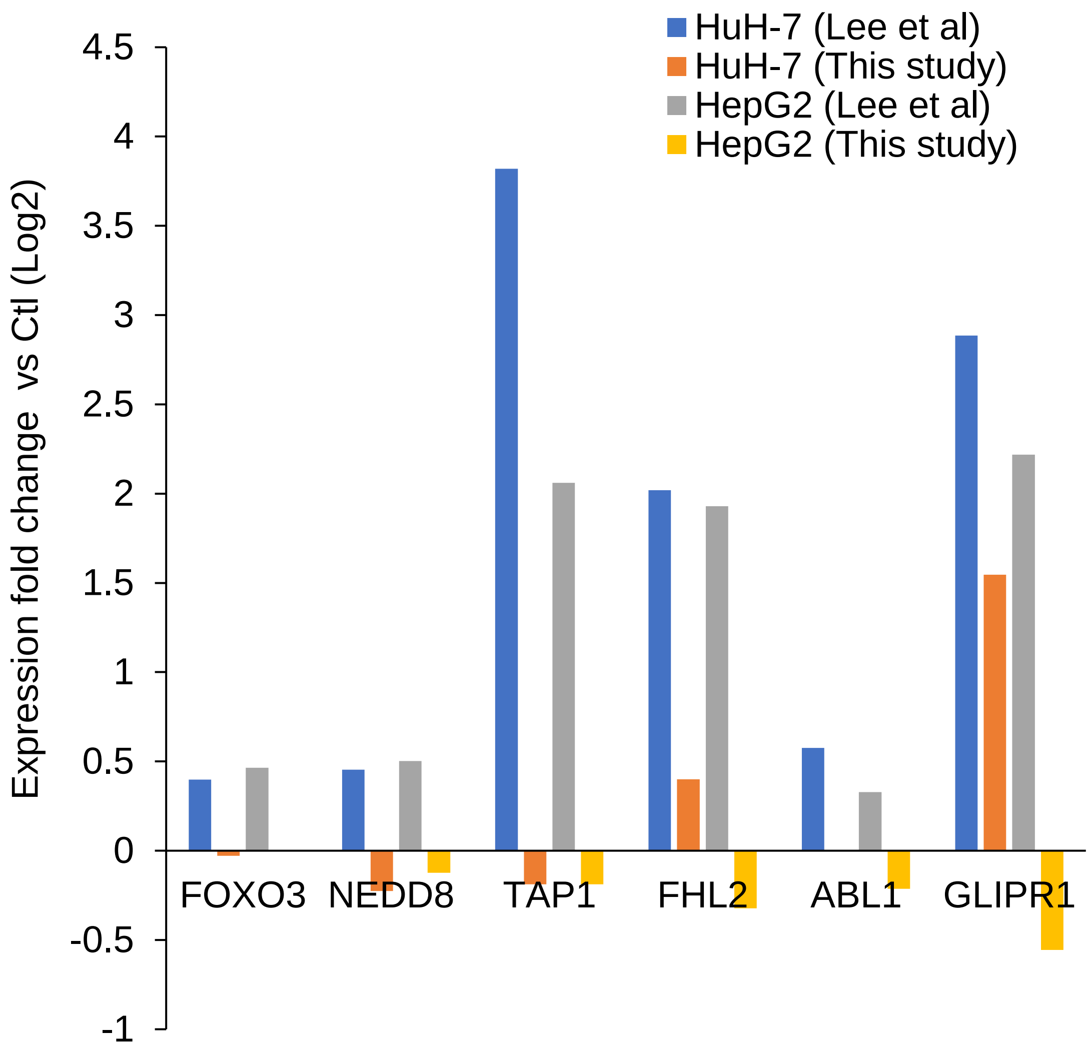

Figure S11. Impact of COP1 silencing on a subset of genes linked to p53 regulation. Transcript abundance changes as a result of COP1 silencing. Gene selection is by Lee et al. Values from both studies are shown.
