# Supplementary material for: Distinct roles of Constitutive Photomorphogenesis Protein 1 homolog (COP1) in human hepatocyte models
Source: Front Mol Biosci. 2025 Feb 7;12:1548582. doi: 10.3389/fmolb.2025.1548582 (PMC11842253; doi:10.3389/fmolb.2025.1548582)
Supplement: Supplementary file 2 [file DataSheet3.pdf]

SUPPLEMENTARY TABLES

Enrichment analysis by Overerepresentation analysis (ORA) and Gene Set Enrichment analyses (GSEA). NCBI Entrez Genes were mapped via WebGestalt. GSEA was performed using the complete transcriptome fold-change dataset. Nominally changed transcripts were used for ORA. Default parameters were used. FDR significant hits (q < 0.05) hits are shown

**Table S1**	Over-representation of HepG2 and HuH-7 shared hits.
**Table S2**	ORA analyses of nominally changed transcripts in HepG2, HuH7 and Primary Hepatocytes
**Table S3**	ORA analyses using 3 randomized ID inputs
**Table S4**	Gene Set Enrichment analyses of transcriptome impacts of COP1 suppression in HuH-7, HepG2 and Hepatocytes
**Table S5**	Ingenuity Canonical Pathways identified in COP1 suppressed cells
**Table S6**	Upstream regulators predicted in at least one cell type by IPA upstream regulators
**Table S7**	Over-representation analyses of COP1 and HNF4A targeted hits
**Table S8**	Comparison of Gene list from Lee et al (Suppl Table 2) with values from the current study.

Table S1. ORA React, KEGG and GO of HepG2 and HuH-7 shared hits. Nominally significant hits common to HepG2 and HuH-7 cells were analyzed by ORA. Analysis performed via WebGestalt. Only FDR significant hits are shown. Size, Gene Set Size; Ratio: Enrichment ratio (number of actual hits mapped to input and geneset overlap vs number of hits predicted by chance). P value, nominal pvalue of overlap. FDR, False Discovery Rate.

GO (Biological_Process_noRedundant)					
Gene Set	Description	Size	Ratio	P Value	FDR
GO:0006260	DNA replication	259	6.4	1.1E-15	9.4E-13
GO:0044843	cell cycle G1/S phase transition	236	4.9	5.3E-09	2.3E-06
GO:0044772	mitotic cell cycle phase transition	464	3.3	3.8E-08	1.1E-05
GO:0071103	DNA conformation change	230	4.0	2.7E-06	5.7E-04
GO:0006301	postreplication repair	47	8.6	1.5E-05	2.6E-03
GO:0000075	cell cycle checkpoint	211	3.5	8.2E-05	1.2E-02
GO:0010948	negative regulation of cell cycle process	266	3.0	2.3E-04	2.8E-02
GO:0003170	heart valve development	52	6.6	2.7E-04	2.9E-02
GO:0071897	DNA biosynthetic process	184	3.4	3.8E-04	3.0E-02
GO:0002065	columnar/cuboidal epithelial cell differentiation	102	4.5	4.0E-04	3.0E-02
GO:0042769	DNA damage response, detection of DNA damag	37	7.8	4.2E-04	3.0E-02
GO:0031589	cell-substrate adhesion	316	2.7	4.3E-04	3.0E-02
GO:0022616	DNA strand elongation	23	10.0	6.1E-04	4.0E-02
GO:0048285	organelle fission	444	2.3	7.7E-04	4.4E-02
GO:1901342	regulation of vasculature development	300	2.7	7.8E-04	4.4E-02
KEGG					
Gene Set	Description	Size	Ratio	P Value	FDR
hsa04110	Cell cycle	119	6.5	2.3E-08	7.6E-06
hsa03030	DNA replication	34	13.0	1.1E-07	1.8E-05
REACTOME					
Gene Set	Description	Size	Ratio	P Value	FDR
R-HSA-176974	Unwinding of DNA	12	41.3	3.7E-14	6.4E-11
R-HSA-69190	DNA strand elongation	31	19.5	3.3E-12	2.8E-09
R-HSA-176187	Activation of ATR in response to replication stre	37	16.4	3.0E-11	1.7E-08
R-HSA-69306	DNA Replication	124	7.5	7.8E-11	3.3E-08
R-HSA-69239	Synthesis of DNA	116	7.6	2.6E-10	8.9E-08
R-HSA-69242	S Phase	155	6.4	3.5E-10	9.9E-08
R-HSA-1640170	Cell Cycle	609	3.2	8.3E-10	1.8E-07
R-HSA-453279	Mitotic G1-G1/S phases	143	6.5	7.6E-10	1.8E-07
R-HSA-69206	G1/S Transition	128	6.9	1.2E-09	2.2E-07
R-HSA-68962	Activation of the pre-replicative complex	33	15.0	4.7E-09	8.0E-07
R-HSA-69620	Cell Cycle Checkpoints	280	4.3	6.6E-09	1.0E-06
R-HSA-69278	Cell Cycle, Mitotic	507	3.1	3.2E-08	4.6E-06
R-HSA-539107	Activation of E2F1 target genes at G1/S	28	13.8	5.0E-07	6.1E-05
R-HSA-69205	G1/S-Specific Transcription	28	13.8	5.0E-07	6.1E-05
R-HSA-69002	DNA Replication Pre-Initiation	84	6.5	2.7E-06	3.0E-04
R-HSA-68867	Assembly of the pre-replicative complex	67	7.4	3.1E-06	3.3E-04

R-HSA-69052	Switching of origins to a post-replicative state	88	6.3	4.1E-06	4.1E-04
R-HSA-68949	Orc1 removal from chromatin	70	7.1	4.5E-06	4.2E-04
R-HSA-1538133	G0 and Early G1	27	12.2	7.2E-06	6.1E-04
R-HSA-69481	G2/M Checkpoints	161	4.4	7.1E-06	6.1E-04
R-HSA-73893	DNA Damage Bypass	48	8.0	2.3E-05	1.9E-03
R-HSA-68689	CDC6 association with the ORC:origin complex	11	20.0	3.2E-05	2.4E-03
R-HSA-73894	DNA Repair	302	2.9	1.2E-04	9.0E-03
R-HSA-5685942	HDR through Homologous Recombination (HRR)	64	6.0	1.5E-04	1.1E-02
R-HSA-110314	Recognition of DNA damage by PCNA-containing	30	9.2	1.9E-04	1.3E-02
R-HSA-5656169	Termination of translesion DNA synthesis	31	8.9	2.2E-04	1.4E-02
R-HSA-1362277	Transcription of E2F targets under negative cont	19	11.6	3.3E-04	2.1E-02
R-HSA-5693567	HDR through Homologous Recombination (HRR)	123	4.0	4.0E-04	2.4E-02
R-HSA-110313	Translesion synthesis by Y family DNA polymeras	38	7.2	5.8E-04	3.2E-02
R-HSA-5693538	Homology Directed Repair	129	3.8	5.6E-04	3.2E-02
R-HSA-5693616	Presynaptic phase of homologous DNA pairing a	38	7.2	5.8E-04	3.2E-02
R-HSA-5693579	Homologous DNA Pairing and Strand Exchange	41	6.7	8.3E-04	4.4E-02

Table S2. ORA analyses of nominally changed transcripts in HepG2, HuH7 and Primary Hepatocytes. Over-representation analysis using Gene Ontology ("Biological_Process_noRedundant"), as well as KEGG and Reactome pathways datasets. Listed by increasing FDR values. Hits common to more than one cell type are highlighted in red.

GO

HuH-7

Gene Set	Description	FDR
GO:0006260	DNA replication	3.7E-10
GO:0044772	mitotic cell cycle phase transition	4.9E-06
GO:0000075	cell cycle checkpoint	1.1E-05
GO:0044843	cell cycle G1/S phase transition	2.7E-05
GO:0071103	DNA conformation change	2.1E-04
GO:0022616	DNA strand elongation	5.1E-04
GO:1904888	cranial skeletal system development	1.2E-03
GO:0006302	double-strand break repair	1.2E-02
GO:0001525	angiogenesis	1.2E-02

HepG2

Gene Set	Description	FDR
GO:0044282	small molecule catabolic process	4.5E-10
GO:0006631	fatty acid metabolic process	2.2E-06
GO:0006260	DNA replication	1.7E-05
GO:0006732	coenzyme metabolic process	2.2E-04
GO:1901615	organic hydroxy compound metabolic p	4.4E-04
GO:0002576	platelet degranulation	4.4E-04
GO:0002526	acute inflammatory response	6.5E-04
GO:0072376	protein activation cascade	7.6E-04
GO:0051604	protein maturation	8.1E-04
GO:0019216	regulation of lipid metabolic process	1.1E-03
GO:0006790	sulfur compound metabolic process	1.1E-03
GO:0016053	organic acid biosynthetic process	1.2E-03
GO:0055088	lipid homeostasis	2.5E-03
GO:0008202	steroid metabolic process	2.7E-03
GO:0006520	cellular amino acid metabolic process	3.6E-03
GO:2000147	positive regulation of cell motility	4.2E-03
GO:0051701	interaction with host	6.8E-03
GO:0006979	response to oxidative stress	8.2E-03
GO:0031589	cell-substrate adhesion	1.1E-02
GO:0001525	angiogenesis	1.3E-02
GO:0050817	coagulation	1.4E-02
GO:0048732	gland development	1.4E-02
GO:0019058	viral life cycle	1.4E-02

GO:0014812	muscle cell migration	1.5E-02
GO:0061008	hepaticobiliary system development	1.5E-02
GO:0009636	response to toxic substance	1.6E-02
GO:0006638	neutral lipid metabolic process	1.7E-02
GO:0051188	cofactor biosynthetic process	1.8E-02
GO:0010038	response to metal ion	1.8E-02
GO:0046434	organophosphate catabolic process	1.8E-02
GO:0042558	pteridine-containing compound metabo	1.8E-02
GO:0048545	response to steroid hormone	1.9E-02
GO:0033865	nucleoside bisphosphate metabolic pro	2.0E-02
GO:0042737	drug catabolic process	2.0E-02
GO:0001101	response to acid chemical	2.0E-02
GO:0016042	lipid catabolic process	2.0E-02
GO:0050727	regulation of inflammatory response	2.1E-02
GO:0006090	pyruvate metabolic process	2.1E-02
GO:0034404	nucleobase-containing small molecule	2.1E-02
GO:0044772	mitotic cell cycle phase transition	2.1E-02
GO:0015849	organic acid transport	2.8E-02
GO:1901342	regulation of vasculature development	3.8E-02
GO:0032103	positive regulation of response to exter	3.8E-02
GO:1903034	regulation of response to wounding	4.5E-02

Hepatocytes

Gene Set	Description	FDR
GO:0006631	fatty acid metabolic process	1.1E-06
GO:0044282	small molecule catabolic process	7.8E-06
GO:0015711	organic anion transport	8.0E-06
GO:1901615	organic hydroxy compound metabolic p	8.0E-06
GO:0010876	lipid localization	1.5E-05
GO:0015849	organic acid transport	1.5E-05
GO:0008202	steroid metabolic process	4.6E-05
GO:0055088	lipid homeostasis	9.3E-05
GO:1901568	fatty acid derivative metabolic process	1.2E-04
GO:0050878	regulation of body fluid levels	2.0E-04
GO:0050673	epithelial cell proliferation	3.9E-04
GO:0007586	digestion	3.9E-04
GO:0015850	organic hydroxy compound transport	4.1E-04
GO:0016053	organic acid biosynthetic process	4.1E-04
GO:0048732	gland development	4.8E-04
GO:0006790	sulfur compound metabolic process	1.8E-03
GO:0097164	ammonium ion metabolic process	2.0E-03
GO:0062012	regulation of small molecule metabolic	4.2E-03
GO:0010038	response to metal ion	4.7E-03
GO:0002449	lymphocyte mediated immunity	4.7E-03
GO:0006959	humoral immune response	4.7E-03

GO:0019216	regulation of lipid metabolic process	4.7E-03
GO:0009410	response to xenobiotic stimulus	4.7E-03
GO:0042445	hormone metabolic process	5.1E-03
GO:0043491	protein kinase B signaling	5.1E-03
GO:0042737	drug catabolic process	5.1E-03
GO:0009112	nucleobase metabolic process	5.1E-03
GO:0006575	cellular modified amino acid metabolic	6.4E-03
GO:0006732	coenzyme metabolic process	7.6E-03
GO:0043062	extracellular structure organization	9.4E-03
GO:0032528	microvillus organization	1.1E-02
GO:0070555	response to interleukin-1	1.2E-02
GO:0001655	urogenital system development	1.4E-02
GO:0007015	actin filament organization	1.4E-02
GO:0015748	organophosphate ester transport	1.4E-02
GO:0014812	muscle cell migration	1.4E-02
GO:0071241	cellular response to inorganic substanc	1.5E-02
GO:0016042	lipid catabolic process	1.5E-02
GO:0002576	platelet degranulation	1.5E-02
GO:0009636	response to toxic substance	1.5E-02
GO:0050817	coagulation	1.9E-02
GO:0072376	protein activation cascade	2.1E-02
GO:0070371	ERK1 and ERK2 cascade	2.1E-02
GO:0003013	circulatory system process	2.9E-02
GO:0031667	response to nutrient levels	3.8E-02
GO:0072523	purine-containing compound catabolic	3.9E-02
GO:0051188	cofactor biosynthetic process	4.5E-02
GO:0090130	tissue migration	4.8E-02
GO:0046486	glycerolipid metabolic process	4.9E-02

KEGG

HuH-7

Gene Set	Description	FDR
hsa04110	Cell cycle	1.0E-07
hsa03030	DNA replication	3.9E-06
hsa03430	Mismatch repair	2.7E-02

HepG2

Gene Set	Description	FDR
hsa01100	Metabolic pathways	5.7E-07
hsa04610	Complement and coagulation cascade	5.0E-06
hsa00100	Steroid biosynthesis	7.3E-04
hsa03030	DNA replication	2.0E-02
hsa00480	Glutathione metabolism	3.6E-02
hsa04975	Fat digestion and absorption	3.9E-02

Hepatocytes

Gene Set	Description	FDR
hsa00982	Drug metabolism	8.6E-07
hsa05204	Chemical carcinogenesis	2.8E-06
hsa00830	Retinol metabolism	4.3E-06
hsa00980	Metabolism of xenobiotics by cytochror	4.3E-06
hsa00140	Steroid hormone biosynthesis	4.0E-05
hsa00983	Drug metabolism	4.0E-05
hsa00071	Fatty acid degradation	2.0E-03
hsa00340	Histidine metabolism	2.0E-03
hsa04978	Mineral absorption	3.0E-03
hsa00053	Ascorbate and aldarate metabolism	4.5E-03
hsa04610	Complement and coagulation cascade	7.1E-03
hsa03320	PPAR signaling pathway	1.1E-02
hsa00040	Pentose and glucuronate interconversi	1.1E-02
hsa00380	Tryptophan metabolism	1.3E-02
hsa01212	Fatty acid metabolism	2.5E-02
hsa00410	beta-Alanine metabolism	2.5E-02
hsa00480	Glutathione metabolism	2.5E-02
hsa01100	Metabolic pathways	2.5E-02

REACTOME

HuH-7

Gene Set	Description	FDR
R-HSA-68962	Activation of the pre-replicative comple	4.68E-11
R-HSA-176187	Activation of ATR in response to replic	4.68E-11
R-HSA-69306	DNA Replication	1.21E-09
R-HSA-69190	DNA strand elongation	1.31E-08
R-HSA-1640170	Cell Cycle	1.78E-08
R-HSA-69278	Cell Cycle, Mitotic	1.84E-07
R-HSA-176974	Unwinding of DNA	1.84E-07
R-HSA-453279	Mitotic G1-G1/S phases	1.84E-07
R-HSA-69620	Cell Cycle Checkpoints	1.44E-06
R-HSA-69206	G1/S Transition	1.54E-06
R-HSA-69239	Synthesis of DNA	1.75E-06
R-HSA-69242	S Phase	4.43E-06
R-HSA-69002	DNA Replication Pre-Initiation	2.48E-05
R-HSA-69481	G2/M Checkpoints	2.35E-03
R-HSA-1538133	G0 and Early G1	3.58E-03
R-HSA-73886	Chromosome Maintenance	3.58E-03
R-HSA-539107	Activation of E2F1 target genes at G1/	4.25E-03
R-HSA-69205	G1/S-Specific Transcription	4.25E-03
R-HSA-5693538	Homology Directed Repair	4.25E-03
R-HSA-174417	Telomere C-strand (Lagging Strand) S	4.25E-03

R-HSA-180786	Extension of Telomeres	5.49E-03
R-HSA-68689	CDC6 association with the ORC:origin	7.94E-03
R-HSA-5651801	PCNA-Dependent Long Patch Base Ex	8.17E-03
R-HSA-73894	DNA Repair	2.15E-02
R-HSA-72649	Translation initiation complex formation	2.45E-02
R-HSA-5693532	DNA Double-Strand Break Repair	2.50E-02
R-HSA-156827	L13a-mediated translational silencing c	2.50E-02
R-HSA-72662	Activation of the mRNA upon binding o	2.50E-02
R-HSA-72702	Ribosomal scanning and start codon re	2.50E-02
R-HSA-5693567	HDR through Homologous Recombina	2.50E-02
R-HSA-5696397	Gap-filling DNA repair synthesis and lig	2.62E-02
R-HSA-110314	Recognition of DNA damage by PCNA	2.74E-02
R-HSA-69186	Lagging Strand Synthesis	2.79E-02
R-HSA-72706	GTP hydrolysis and joining of the 60S	2.79E-02
R-HSA-5685942	HDR through Homologous Recombina	3.01E-02
R-HSA-110373	Resolution of AP sites via the multiple-	3.08E-02
R-HSA-69052	Switching of origins to a post-replicative	3.43E-02
R-HSA-73884	Base Excision Repair	3.43E-02
R-HSA-73933	Resolution of Abasic Sites (AP sites)	3.43E-02
R-HSA-5693616	Presynaptic phase of homologous DNA	3.43E-02

HepG2

Gene Set	Description	FDR
R-HSA-1430728	Metabolism	9.7E-07
R-HSA-176974	Unwinding of DNA	8.4E-05
R-HSA-69190	DNA strand elongation	1.1E-04
R-HSA-114608	Platelet degranulation	7.4E-04
R-HSA-1640170	Cell Cycle	1.3E-03
R-HSA-539107	Activation of E2F1 target genes at G1/S	1.3E-03
R-HSA-69205	G1/S-Specific Transcription	1.3E-03
R-HSA-76002	Platelet activation, signaling and aggre	1.3E-03
R-HSA-76005	Response to elevated platelet cytosolic	1.3E-03
R-HSA-69278	Cell Cycle, Mitotic	1.4E-03
R-HSA-109582	Hemostasis	1.5E-03
R-HSA-191273	Cholesterol biosynthesis	2.5E-03
R-HSA-977606	Regulation of Complement cascade	2.5E-03
R-HSA-166658	Complement cascade	3.3E-03
R-HSA-6788656	Histidine, lysine, phenylalanine, tyrosin	5.1E-03
R-HSA-174577	Activation of C3 and C5	5.8E-03
R-HSA-211859	Biological oxidations	6.2E-03
R-HSA-556833	Metabolism of lipids	1.1E-02
R-HSA-8957275	Post-translational protein phosphorylat	1.2E-02
R-HSA-8978868	Fatty acid metabolism	1.5E-02
R-HSA-69620	Cell Cycle Checkpoints	1.5E-02
R-HSA-75205	Dissolution of Fibrin Clot	2.8E-02
R-HSA-1538133	G0 and Early G1	3.5E-02

R-HSA-8957322	Metabolism of steroids	3.5E-02
R-HSA-381426	Regulation of Insulin-like Growth Facto	4.4E-02
R-HSA-174411	Polymerase switching on the C-strand	4.8E-02
R-HSA-69091	Polymerase switching	4.8E-02
R-HSA-69109	Leading Strand Synthesis	4.8E-02

Hepatocytes

Gene Set	Description	FDR
R-HSA-211859	Biological oxidations	6.3E-10
R-HSA-1430728	Metabolism	3.2E-06
R-HSA-156580	Phase II - Conjugation of compounds	7.6E-06
R-HSA-211945	Phase I - Functionalization of compour	4.3E-04
R-HSA-556833	Metabolism of lipids	9.5E-04
R-HSA-3000171	Non-integrin membrane-ECM interactic	2.8E-03
R-HSA-8963899	Plasma lipoprotein remodeling	2.8E-03
R-HSA-211958	Miscellaneous substrates	4.2E-03
R-HSA-211897	Cytochrome P450 - arranged by substr	6.4E-03
R-HSA-211981	Xenobiotics	1.2E-02
R-HSA-156588	Glucuronidation	1.2E-02
R-HSA-5660526	Response to metal ions	1.2E-02
R-HSA-8978868	Fatty acid metabolism	1.2E-02
R-HSA-3000170	Syndecan interactions	1.2E-02
R-HSA-5661231	Metallothioneins bind metals	1.2E-02
R-HSA-211935	Fatty acids	1.8E-02
R-HSA-2142753	Arachidonic acid metabolism	1.8E-02
R-HSA-211979	Eicosanoids	2.1E-02
R-HSA-174824	Plasma lipoprotein assembly, remodeli	2.1E-02
R-HSA-8963889	Assembly of active LPL and LIPC lipas	2.7E-02

Table S3. ORA analyses using 3 randomized ID inputs from . Over-representation analysis using KEGG and Reactome terms was performed on 3 lists consisting 4262 randomized IDs from the HuGene 2.0 array. Listed by increasing FDR values. Hits shared by more than one list are highlighted in red (KEGG) or yellow (Reactome).

KEGG

Set 1

Gene Set	Description	FDR
hsa04612	Antigen processing and presentation	4.6E-05
hsa05332	Graft-versus-host disease	1.2E-03
hsa05330	Allograft rejection	1.4E-03
hsa04940	Type I diabetes mellitus	4.0E-03
hsa05310	Asthma	1.0E-02
hsa05145	Toxoplasmosis	1.0E-02
hsa05320	Autoimmune thyroid disease	1.0E-02
hsa04145	Phagosome	1.6E-02
hsa05416	Viral myocarditis	1.8E-02
hsa00500	Starch and sucrose metabolism	1.8E-02
hsa05322	Systemic lupus erythematosus	7.5E-02
hsa05150	Staphylococcus aureus infection	7.7E-02
hsa05166	Human T-cell leukemia virus 1 infection	1.5E-01
hsa05323	Rheumatoid arthritis	1.9E-01
hsa04672	Intestinal immune network for IgA production	1.9E-01
hsa04213	Longevity regulating pathway	2.5E-01
hsa05168	Herpes simplex infection	3.1E-01
hsa04914	Progesterone-mediated oocyte maturation	3.1E-01
hsa04640	Hematopoietic cell lineage	3.2E-01
hsa04218	Cellular senescence	3.3E-01

Set 2

Gene Set	Description	FDR
hsa04612	Antigen processing and presentation	4.0E-04
hsa05332	Graft-versus-host disease	4.0E-04
hsa05330	Allograft rejection	2.9E-03
hsa05416	Viral myocarditis	4.3E-03
hsa04940	Type I diabetes mellitus	5.7E-03
hsa05320	Autoimmune thyroid disease	1.7E-02
hsa05310	Asthma	1.8E-02
hsa04672	Intestinal immune network for IgA production	5.7E-02
hsa04650	Natural killer cell mediated cytotoxicity	8.9E-02
hsa05168	Herpes simplex infection	9.4E-02
hsa05164	Influenza A	1.0E-01
hsa04640	Hematopoietic cell lineage	1.0E-01
hsa05166	Human T-cell leukemia virus 1 infection	2.0E-01

hsa01521	EGFR tyrosine kinase inhibitor resistance	2.1E-01
hsa03015	mRNA surveillance pathway	2.1E-01
hsa04145	Phagosome	2.3E-01
hsa04064	NF-kappa B signaling pathway	2.6E-01
hsa04977	Vitamin digestion and absorption	3.2E-01
hsa05145	Toxoplasmosis	3.9E-01
hsa05219	Bladder cancer	3.9E-01

Set 3

Gene Set	Description	FDR
hsa04940	Type I diabetes mellitus	1.4E-02
hsa03008	Ribosome biogenesis in eukaryotes	6.7E-02
hsa05310	Asthma	7.1E-02
hsa05322	Systemic lupus erythematosus	7.1E-02
hsa05330	Allograft rejection	7.1E-02
hsa05169	Epstein-Barr virus infection	2.9E-01
hsa04714	Thermogenesis	2.9E-01
hsa05332	Graft-versus-host disease	2.9E-01
hsa05140	Leishmaniasis	3.6E-01
hsa05012	Parkinson disease	3.6E-01
hsa04926	Relaxin signaling pathway	5.2E-01
hsa00190	Oxidative phosphorylation	5.2E-01
hsa05145	Toxoplasmosis	5.6E-01
hsa05321	Inflammatory bowel disease (IBD)	5.6E-01
hsa04659	Th17 cell differentiation	5.6E-01
hsa01523	Antifolate resistance	5.6E-01
hsa04260	Cardiac muscle contraction	5.6E-01
hsa04612	Antigen processing and presentation	5.9E-01
hsa05320	Autoimmune thyroid disease	6.8E-01
hsa05168	Herpes simplex infection	6.8E-01

REACTOME

set1

Gene Set	Description	FDR
R-HSA-189085	Digestion of dietary carbohydrate	4.7E-01
R-HSA-3371568	Attenuation phase	7.5E-01
R-HSA-202430	Translocation of ZAP-70 to Immunological synapse	7.5E-01
R-HSA-8935690	Digestion	7.5E-01
R-HSA-3560782	Diseases associated with glycosaminoglycan metabolism	7.5E-01
R-HSA-3371571	HSF1-dependent transactivation	7.5E-01
R-HSA-202427	Phosphorylation of CD3 and TCR zeta chains	7.5E-01
R-HSA-174577	Activation of C3 and C5	7.5E-01
R-HSA-3656225	Defective CHST6 causes MCDC1	7.5E-01
R-HSA-3656243	Defective ST3GAL3 causes MCT12 and EIEE15	7.5E-01
R-HSA-3656244	Defective B4GALT1 causes B4GALT1-CDG (CDG-2d)	7.5E-01
R-HSA-389948	PD-1 signaling	7.7E-01

R-HSA-2129379	Molecules associated with elastic fibres	1.0E+00
R-HSA-8963743	Digestion and absorption	1.0E+00
R-HSA-2022854	Keratan sulfate biosynthesis	1.0E+00
R-HSA-877300	Interferon gamma signaling	1.0E+00
R-HSA-5603029	IkBA variant leads to EDA-ID	1.0E+00
R-HSA-3371453	Regulation of HSF1-mediated heat shock response	1.0E+00
R-HSA-446219	Synthesis of substrates in N-glycan biosythesis	1.0E+00
R-HSA-446193	Biosynthesis of the N-glycan precursor (dolichol lipid-linked ol	1.0E+00

set2

Gene Set	Description	FDR
R-HSA-1181150	Signaling by NODAL	7.8E-01
R-HSA-1236975	Antigen processing-Cross presentation	1.0E+00
R-HSA-1236977	Endosomal/Vacuolar pathway	1.0E+00
R-HSA-1433617	Regulation of signaling by NODAL	1.0E+00
R-HSA-1643685	Disease	1.0E+00
R-HSA-202427	Phosphorylation of CD3 and TCR zeta chains	7.8E-01
R-HSA-202430	Translocation of ZAP-70 to Immunological synapse	1.0E+00
R-HSA-2187335	The retinoid cycle in cones (daylight vision)	5.8E-01
R-HSA-2428928	IRS-related events triggered by IGF1R	1.0E+00
R-HSA-352230	Amino acid transport across the plasma membrane	1.0E+00
R-HSA-376176	Signaling by ROBO receptors	1.0E+00
R-HSA-389948	PD-1 signaling	1.0E+00
R-HSA-444257	RSK activation	1.0E+00
R-HSA-5579029	Metabolic disorders of biological oxidation enzymes	7.8E-01
R-HSA-5676594	TNF receptor superfamily (TNFSF) members mediating non-ca	7.8E-01
R-HSA-877300	Interferon gamma signaling	7.8E-01
R-HSA-8866910	TFAP2 (AP-2) family regulates transcription of growth factors	9.9E-01
R-HSA-9010553	Regulation of expression of SLITs and ROBOs	1.0E+00
R-HSA-927802	Nonsense-Mediated Decay (NMD)	7.8E-01
R-HSA-975957	Nonsense Mediated Decay (NMD) enhanced by the Exon Junc	7.8E-01

set3

Gene Set	Description	FDR
R-HSA-5689880	Ub-specific processing proteases	3.7E-02
R-HSA-389948	PD-1 signaling	3.9E-02
R-HSA-202424	Downstream TCR signaling	3.9E-02
R-HSA-202403	TCR signaling	9.5E-02
R-HSA-5688426	Deubiquitination	2.7E-01
R-HSA-202427	Phosphorylation of CD3 and TCR zeta chains	2.7E-01
R-HSA-388841	Costimulation by the CD28 family	2.7E-01
R-HSA-202430	Translocation of ZAP-70 to Immunological synapse	4.5E-01
R-HSA-392499	Metabolism of proteins	9.9E-01
R-HSA-1169091	Activation of NF-kappaB in B cells	9.9E-01
R-HSA-2871837	FCERI mediated NF-kB activation	9.9E-01
R-HSA-1428517	The citric acid (TCA) cycle and respiratory electron transport	9.9E-01
R-HSA-163200	Respiratory electron transport, ATP synthesis by chemiosmoti	1.0E+00

R-HSA-597592	Post-translational protein modification	1.0E+00
R-HSA-2262752	Cellular responses to stress	1.0E+00
R-HSA-1280218	Adaptive Immune System	1.0E+00
R-HSA-202433	Generation of second messenger molecules	1.0E+00
R-HSA-5676590	NIK-->noncanonical NF-kB signaling	1.0E+00
R-HSA-5684264	MAP3K8 (TPL2)-dependent MAPK1/3 activation	1.0E+00
R-HSA-977443	GABA receptor activation	1.0E+00

Table S4. Gene Set Enrichment analyses of transcriptome impacts of COP1 suppression in HuH-7, HepG2 and Hepatocytes. Transcripts were mapped to Gene Ontology (geneontology_Biological_Process_noRedundant) and KEGG categories. Arranged by Normalized Effect Size. Hits common to more than one cell type are highlighted in red.

GO

HuH-7

Gene Set	Description	NES	FDR
GO:0006260	DNA replication	-4.4341	0
GO:0044772	mitotic cell cycle phase transition	-3.9099	0
GO:0044839	cell cycle G2/M phase transition	-3.519	0
GO:0044843	cell cycle G1/S phase transition	-3.382	0
GO:1901987	regulation of cell cycle phase transition	-3.2648	0
GO:0007059	chromosome segregation	-3.2585	0
GO:0000075	cell cycle checkpoint	-3.2432	0
GO:0048285	organelle fission	-3.1988	0
GO:0071103	DNA conformation change	-3.101	0
GO:0010948	negative regulation of cell cycle process	-3.0814	0
GO:0061641	CENP-A containing chromatin organizatic	-3.0146	0
GO:0045930	negative regulation of mitotic cell cycle	-3.0021	0
GO:0051321	meiotic cell cycle	-2.9098	0
GO:0006302	double-strand break repair	-2.792	6.1E-05
GO:0022616	DNA strand elongation	-2.7916	5.7E-05
GO:0006310	DNA recombination	-2.7283	5.4E-05
GO:0006333	chromatin assembly or disassembly	-2.6492	1.0E-04
GO:0071824	protein-DNA complex subunit organizatio	-2.5293	5.7E-04
GO:0034502	protein localization to chromosome	-2.4145	1.4E-03
GO:0051052	regulation of DNA metabolic process	-2.3984	1.8E-03
GO:0070988	demethylation	2.2281	3.8E-02
GO:0070972	protein localization to endoplasmic reticul	2.2503	3.6E-02
GO:0032941	secretion by tissue	2.2572	4.2E-02
GO:0098742	cell-cell adhesion via plasma-membrane a	2.2906	4.2E-02
GO:0044262	cellular carbohydrate metabolic process	2.3058	4.9E-02

HepG2

Gene Set	Description	NES	FDR
GO:0044282	small molecule catabolic process	-3.9555	0
GO:0006631	fatty acid metabolic process	-3.7902	0
GO:0008202	steroid metabolic process	-3.6309	0
GO:0072376	protein activation cascade	-3.6281	0
GO:0055088	lipid homeostasis	-3.4617	0
GO:1901615	organic hydroxy compound metabolic pro	-3.2933	0
GO:0006091	generation of precursor metabolites and e	-3.2223	0
GO:0016042	lipid catabolic process	-3.2098	0
GO:0006732	coenzyme metabolic process	-3.142	0

GO:0016053	organic acid biosynthetic process	-3.1208	0
GO:0010876	lipid localization	-3.0669	0
GO:0006638	neutral lipid metabolic process	-3.0618	0
GO:0016999	antibiotic metabolic process	-2.98	0
GO:0042180	cellular ketone metabolic process	-2.9127	0
GO:0006520	cellular amino acid metabolic process	-2.8963	0
GO:0002526	acute inflammatory response	-2.8891	0
GO:0015711	organic anion transport	-2.8479	0
GO:0033865	nucleoside bisphosphate metabolic proce	-2.8237	0
GO:0006575	cellular modified amino acid metabolic pr	-2.7705	0
GO:0015748	organophosphate ester transport	-2.7459	0
GO:0071825	protein-lipid complex subunit organizatior	-2.6738	8.1E-05
GO:0043574	peroxisomal transport	-2.6361	1.1E-04
GO:0009259	ribonucleotide metabolic process	-2.5247	5.3E-04
GO:0034976	response to endoplasmic reticulum stress	-2.5032	5.8E-04
GO:0042737	drug catabolic process	-2.4131	1.3E-03
GO:0070085	glycosylation	-2.3514	2.4E-03
GO:0009141	nucleoside triphosphate metabolic proces	-2.2785	3.2E-03
GO:0140053	mitochondrial gene expression	-2.2455	3.6E-03
GO:0010257	NADH dehydrogenase complex assembly	-2.1179	8.2E-03
GO:0044872	lipoprotein localization	-2.1151	8.2E-03
GO:0035456	response to interferon-beta	-2.0008	1.6E-02
GO:0072337	modified amino acid transport	-1.933	2.3E-02
GO:0006081	cellular aldehyde metabolic process	-1.9249	2.4E-02
GO:0046486	glycerolipid metabolic process	-1.8947	2.7E-02
GO:1903513	endoplasmic reticulum to cytosol transpo	-1.8833	2.8E-02
GO:0009743	response to carbohydrate	-1.8483	3.3E-02
GO:0001101	response to acid chemical	-1.8227	3.7E-02
GO:0046939	nucleotide phosphorylation	-1.8	4.2E-02
GO:0014075	response to amine	1.8837	4.3E-02
GO:0050867	positive regulation of cell activation	1.8942	4.2E-02
GO:0050906	detection of stimulus involved in sensory	1.911	3.9E-02
GO:0007606	sensory perception of chemical stimulus	1.9188	3.8E-02
GO:0003158	endothelium development	1.9657	3.1E-02
GO:0045930	negative regulation of mitotic cell cycle	2.0053	2.6E-02
GO:1902850	microtubule cytoskeleton organization inv	2.0089	2.5E-02
GO:0021915	neural tube development	2.018	2.5E-02
GO:0051052	regulation of DNA metabolic process	2.0768	2.0E-02
GO:0048285	organelle fission	2.2032	1.2E-02
GO:0001667	ameboidal-type cell migration	2.2073	1.2E-02
GO:0051493	regulation of cytoskeleton organization	2.2178	1.1E-02
GO:0071824	protein-DNA complex subunit organizatio	2.2369	1.0E-02
GO:0090130	tissue migration	2.2429	1.0E-02
GO:0042770	signal transduction in response to DNA d	2.2735	8.1E-03
GO:0032970	regulation of actin filament-based proces	2.3616	4.1E-03
GO:0006302	double-strand break repair	2.3697	4.2E-03
GO:0036297	interstrand cross-link repair	2.37	4.5E-03

GO:2000147	positive regulation of cell motility	2.4025	3.6E-03
GO:0031032	actomyosin structure organization	2.4038	3.8E-03
GO:0061641	CENP-A containing chromatin organizatic	2.4162	3.7E-03
GO:0032200	telomere organization	2.4563	3.2E-03
GO:0071103	DNA conformation change	2.4564	3.5E-03
GO:0006333	chromatin assembly or disassembly	2.474	3.3E-03
GO:0000075	cell cycle checkpoint	2.4782	3.7E-03
GO:0044772	mitotic cell cycle phase transition	2.5351	2.9E-03
GO:0008544	epidermis development	2.5401	3.4E-03
GO:0043588	skin development	2.5598	3.6E-03
GO:0044843	cell cycle G1/S phase transition	2.6259	2.7E-03
GO:0022616	DNA strand elongation	2.6869	1.8E-03
GO:0007059	chromosome segregation	2.8819	5.3E-04
GO:0006260	DNA replication	3.884	0.0E+00

Hepatocytes

Gene Set	Description	NES	FDR
GO:0044282	small molecule catabolic process	-2.5264	2.4E-02
GO:0060840	artery development	-2.4412	2.8E-02
GO:0042180	cellular ketone metabolic process	-2.3657	3.6E-02
GO:0009410	response to xenobiotic stimulus	-2.3339	3.4E-02
GO:0032528	microvillus organization	-2.3277	2.8E-02
GO:0055081	anion homeostasis	-2.2824	3.5E-02
GO:0055088	lipid homeostasis	-2.279	3.0E-02
GO:0008202	steroid metabolic process	-2.242	3.3E-02
GO:0003012	muscle system process	-2.0668	4.8E-02
GO:0015849	organic acid transport	-2.0603	4.8E-02
GO:0009112	nucleobase metabolic process	2.2286	4.0E-02
GO:0018208	peptidyl-proline modification	2.2422	4.9E-02
GO:0006413	translational initiation	2.3506	2.9E-02
GO:0070972	protein localization to endoplasmic reticul	3.4975	0.0E+00

KEGG

HuH-7

Gene Set	Description	NES	FDR
hsa04110	Cell cycle	-4.1587	0
hsa03030	DNA replication	-4.0391	0
hsa03430	Mismatch repair	-2.837	0
hsa03050	Proteasome	-2.8138	0
hsa04914	Progesterone-mediated oocyte maturatio	-2.6523	9.3E-04
hsa03460	Fanconi anemia pathway	-2.6116	9.7E-04
hsa05322	Systemic lupus erythematosus	-2.5167	1.7E-03
hsa04114	Oocyte meiosis	-2.4172	4.1E-03
hsa03008	Ribosome biogenesis in eukaryotes	-2.4085	4.3E-03
hsa05169	Epstein-Barr virus infection	-2.4005	3.9E-03

hsa00480	Glutathione metabolism	-2.3955	3.6E-03
hsa03440	Homologous recombination	-2.3754	3.7E-03
hsa05416	Viral myocarditis	-2.3409	4.6E-03
hsa04721	Synaptic vesicle cycle	-2.2871	5.9E-03
hsa00980	Metabolism of xenobiotics by cytochrome	-2.2784	5.8E-03
hsa05204	Chemical carcinogenesis	-2.1655	1.3E-02
hsa04145	Phagosome	-2.1559	1.3E-02
hsa00190	Oxidative phosphorylation	-2.0401	2.7E-02
hsa05162	Measles	-2.0083	3.2E-02
hsa05203	Viral carcinogenesis	-1.9927	3.4E-02
hsa05110	Vibrio cholerae infection	-1.9627	3.9E-02
hsa05020	Prion diseases	-1.9603	3.8E-02
hsa04218	Cellular senescence	-1.9399	4.2E-02
hsa05034	Alcoholism	-1.9267	4.4E-02
hsa05330	Allograft rejection	-1.9037	4.9E-02

HepG2

Gene Set	Description	NES	FDR
hsa04610	Complement and coagulation cascades	-3.7898	0
hsa04975	Fat digestion and absorption	-3.4699	0
hsa03320	PPAR signaling pathway	-3.4138	0
hsa01100	Metabolic pathways	-3.4032	0
hsa04979	Cholesterol metabolism	-3.1544	0
hsa00100	Steroid biosynthesis	-3.0108	0
hsa01200	Carbon metabolism	-2.8472	1.7E-04
hsa00010	Glycolysis / Gluconeogenesis	-2.8184	1.5E-04
hsa00120	Primary bile acid biosynthesis	-2.7835	1.3E-04
hsa05204	Chemical carcinogenesis	-2.7736	1.2E-04
hsa00071	Fatty acid degradation	-2.7688	1.1E-04
hsa04146	Peroxisome	-2.7052	2.0E-04
hsa01212	Fatty acid metabolism	-2.6842	1.8E-04
hsa00980	Metabolism of xenobiotics by cytochrome	-2.6762	2.5E-04
hsa00190	Oxidative phosphorylation	-2.6735	2.4E-04
hsa01230	Biosynthesis of amino acids	-2.6268	3.0E-04
hsa05150	Staphylococcus aureus infection	-2.5803	4.9E-04
hsa04932	Non-alcoholic fatty liver disease (NAFLD)	-2.5645	4.6E-04
hsa00053	Ascorbate and aldarate metabolism	-2.5204	6.9E-04
hsa04152	AMPK signaling pathway	-2.519	6.5E-04
hsa04976	Bile secretion	-2.4775	8.6E-04
hsa04141	Protein processing in endoplasmic reticul	-2.3154	2.2E-03
hsa05012	Parkinson disease	-2.258	3.4E-03
hsa03050	Proteasome	-2.2135	4.4E-03
hsa03010	Ribosome	-2.1588	5.6E-03
hsa04714	Thermogenesis	-2.1283	6.6E-03
hsa04931	Insulin resistance	-2.0972	8.1E-03
hsa00052	Galactose metabolism	-2.072	9.2E-03

hsa00770	Pantothenate and CoA biosynthesis	-2.0178	1.3E-02
hsa00360	Phenylalanine metabolism	-1.8007	4.5E-02
hsa00510	N-Glycan biosynthesis	-1.7803	4.6E-02
hsa04810	Regulation of actin cytoskeleton	1.915	4.9E-02
hsa04330	Notch signaling pathway	1.9599	3.9E-02
hsa04392	Hippo signaling pathway	1.9612	4.1E-02
hsa04740	Olfactory transduction	1.9686	4.2E-02
hsa03420	Nucleotide excision repair	2.0662	2.4E-02
hsa00531	Glycosaminoglycan degradation	2.0743	2.5E-02
hsa04514	Cell adhesion molecules (CAMs)	2.0766	2.6E-02
hsa03440	Homologous recombination	2.0983	2.5E-02
hsa05202	Transcriptional misregulation in cancer	2.1005	2.7E-02
hsa03430	Mismatch repair	2.1514	2.2E-02
hsa05130	Pathogenic Escherichia coli infection	2.2067	1.7E-02
hsa04218	Cellular senescence	2.2606	1.3E-02
hsa04064	NF-kappa B signaling pathway	2.2664	1.5E-02
hsa03460	Fanconi anemia pathway	2.4478	4.5E-03
hsa03410	Base excision repair	2.4884	4.4E-03
hsa04110	Cell cycle	2.6386	2.0E-03
hsa03030	DNA replication	2.9101	8.2E-04

Hepatocytes

Gene Set	Description	NES	FDR
hsa00980	Metabolism of xenobiotics by cytochrome	-3.4527	0
hsa00982	Drug metabolism	-3.4458	0
hsa00040	Pentose and glucuronate interconversion	-3.3805	0
hsa05204	Chemical carcinogenesis	-3.3029	0
hsa00053	Ascorbate and aldarate metabolism	-3.0858	0
hsa00983	Drug metabolism	-3.039	0
hsa00830	Retinol metabolism	-2.9012	0
hsa00140	Steroid hormone biosynthesis	-2.689	3.3E-04
hsa05206	MicroRNAs in cancer	-2.657	4.4E-04
hsa04928	Parathyroid hormone synthesis, secretior	-2.2627	1.2E-02
hsa04976	Bile secretion	-2.1737	2.0E-02
hsa04270	Vascular smooth muscle contraction	-2.1682	1.9E-02
hsa04964	Proximal tubule bicarbonate reclamation	-2.1256	2.4E-02
hsa00480	Glutathione metabolism	-2.122	2.2E-02
hsa04919	Thyroid hormone signaling pathway	-2.1104	2.2E-02
hsa04530	Tight junction	-2.0869	2.5E-02
hsa04915	Estrogen signaling pathway	-2.0549	2.9E-02
hsa00860	Porphyrin and chlorophyll metabolism	-2.0045	4.0E-02
hsa05219	Bladder cancer	-2.0031	3.8E-02
hsa03320	PPAR signaling pathway	-1.9866	4.0E-02
hsa00590	Arachidonic acid metabolism	-1.9855	3.9E-02
hsa01524	Platinum drug resistance	-1.9552	4.5E-02
hsa04024	cAMP signaling pathway	-1.9547	4.3E-02

hsa00531	Glycosaminoglycan degradation	2.1481	4.6E-02
hsa04978	Mineral absorption	2.6509	1.4E-03
hsa03010	Ribosome	3.4594	0.00

Table S5. Ingenuity Canonical Pathways identified in COP1 suppressed cells . B-H significant pathways are boxed. Only nominally significant hits (green) in at least one cell type are shown. Values are organized as increasing Z-scores, using Hepatocyte values. N/A: no Z-score available for the pathway.

	Hepatocytes	HepG2	HuH7
Xenobiotic Metabolism General Signaling Pathway	-2.921	-3.78	-0.333
Xenobiotic Metabolism PXR Signaling Pathway	-2.655	-5.425	-0.5
Xenobiotic Metabolism CAR Signaling Pathway	-2.596	-4.439	-1
SPINK1 General Cancer Pathway	-2.324	-2.111	-1.342
Complement System	-2.121	-1.941	N/A
Methylglyoxal Degradation III	-2	-2	N/A
CCR3 Signaling in Eosinophils	-1.698	0.832	0.447
Bile Acid Biosynthesis, Neutral Pathway	-1.633	-2.828	N/A
Pregnenolone Biosynthesis	-1.633	N/A	N/A
Nicotine Degradation II	-1.606	-1.291	N/A
Acetone Degradation I (to Methylglyoxal)	-1.508	-0.333	N/A
Aryl Hydrocarbon Receptor Signaling	-1.5	0.728	-1.387
LXR/RXR Activation	-1.461	-5.096	0
Nicotine Degradation III	-1.414	-1.069	N/A
Melatonin Degradation I	-1.342	-1.291	-1
Integrin Signaling	-1.257	3.703	0.258
Thyroid Hormone Metabolism II (via Conjugation and/or Degrada	-1.155	-1.89	N/A
Histidine Degradation VI	-1.134	-1	N/A
PXR/RXR Activation	-1.091	-1.877	-1.265
Bupropion Degradation	-1	-0.333	N/A
STAT3 Pathway	-0.962	0.6	-0.302
Macropinocytosis Signaling	-0.905	2.309	N/A
PPARα/RXRα Activation	-0.898	-1.89	-0.905
Regulation Of The Epithelial Mesenchymal Transition By Growth	-0.73	1.768	0.229
NRF2-mediated Oxidative Stress Response	-0.728	-3.266	0.378
Endothelin-1 Signaling	-0.707	0.192	-0.775
Superpathway of Melatonin Degradation	-0.626	-1.698	-0.447
Estrogen Biosynthesis	-0.577	-0.577	0
Ephrin B Signaling	-0.302	0	N/A
Serotonin Degradation	-0.229	-3.051	N/A
Ephrin Receptor Signaling	-0.209	1	1.265
Kinetochore Metaphase Signaling Pathway	0	1.387	-1
Oxidative Ethanol Degradation III	0	-1.342	N/A
Regulation of Cellular Mechanics by Calpain Protease	0	1.291	0.816
Retinol Biosynthesis	0	-1.732	N/A
Glucose and Glucose-1-phosphate Degradation	0.447	N/A	N/A
IL-6 Signaling	0.6	-0.408	0.378
p38 MAPK Signaling	0.626	0.229	-0.5
Dopamine Degradation	0.707	-2.236	N/A
Phosphatidylglycerol Biosynthesis II (Non-plastidic)	0.707	1	N/A

Purine Nucleotides Degradation II (Aerobic)	0.816	N/A	N/A
Tryptophan Degradation X (Mammalian, via Tryptamine)	0.816	-2.236	N/A
LPS/IL-1 Mediated Inhibition of RXR Function	1.061	2.2	0.333
BER (Base Excision Repair) Pathway	1.342	2.496	-1.508
Guanosine Nucleotides Degradation III	1.342	N/A	N/A
Coagulation System	1.414	0	-1
Acute Phase Response Signaling	1.569	-0.822	0.832
Zymosterol Biosynthesis	2	N/A	N/A
Superpathway of Cholesterol Biosynthesis	2.121	-3.357	N/A
Cholesterol Biosynthesis I	2.449	-2.121	N/A
Cholesterol Biosynthesis II (via 24,25-dihydrolanosterol)	2.449	-2.121	N/A
Cholesterol Biosynthesis III (via Desmosterol)	2.449	-2.121	N/A
Osteoarthritis Pathway	3.042	2.469	-1.414
α-tocopherol Degradation	N/A	N/A	N/A
Atherosclerosis Signaling	N/A	N/A	N/A
Cell Cycle Control of Chromosomal Replication	N/A	3.578	-4.583
Citrulline Biosynthesis	N/A	0	N/A
FXR/RXR Activation	N/A	N/A	N/A
Germ Cell-Sertoli Cell Junction Signaling	N/A	N/A	N/A
Hepatic Cholestasis	N/A	N/A	N/A
IL-12 Signaling and Production in Macrophages	N/A	N/A	N/A
Melatonin Degradation II	N/A	N/A	N/A
Mevalonate Pathway I	N/A	-2.646	N/A
Mismatch Repair in Eukaryotes	N/A	N/A	N/A
Phenylalanine Degradation I (Aerobic)	N/A	0	N/A
Role of BRCA1 in DNA Damage Response	N/A	1	-1.508
Role of Tissue Factor in Cancer	N/A	N/A	N/A
Xenobiotic Metabolism Signaling	N/A	N/A	N/A

Table S6. Upstream regulators predicted in at least one cell type by IPA upstream regulators. Bias-corrected Z-scores are shown incrementally, using hepatocyte values. Negative values, predicted inhibition; positive values, predicted activation. N/A, no Z-score available.

	Hepatocytes	HepG2	HuH7
HNF1A	-3.15	-5.388	1.538
estrogen receptor	-3.035	-1.966	N/A
Growth hormone	-2.976	-1.114	N/A
MRTFB	-2.913	1.73	N/A
ADRB	-2.733	2.014	N/A
NOX4	-2.601	-1.075	N/A
PPARGC1A	-2.572	-4.296	-0.388
OGA	-2.536	-0.126	N/A
F2	-2.523	2.173	N/A
SNAI2	-2.496	0.329	N/A
Tgf beta	-2.474	0.877	N/A
HNF4alpha dimer	-2.429	-2.583	N/A
HSPA5	-2.418	-1.114	N/A
BRD4	-2.395	1.343	-1.515
SRF	-2.378	1.947	-2.563
E2F1	-2.288	3.318	-4.71
RASSF1	-2.253	-0.211	N/A
MRTFA	-2.236	1.693	-1.308
TFAM	-2.236	-1.667	N/A
PROM1	-2.236	1.334	N/A
MOGAT1	-2.213	-2.213	N/A
ACOX1	-2.162	0.071	0.258
IL10RA	-2.147	0.215	-1.789
PPARG	-2.129	-4.328	0.063
GSTO1	-2.129	-0.759	N/A
Alpha catenin	-2.117	-1.809	N/A
RUNX1	-2.071	-0.936	N/A
EDN1	-2.057	2.789	N/A
NR5A1	-2.049	-1.246	-0.113
TGFB1	-2.04	5.721	1.675
ERG	-2.028	3.334	N/A
PKD1	-2.024	-2.805	-2.901
RAC1	-1.945	2.16	N/A
FAS	-1.82	1.331	-2.919
PXR ligand-PXR-Retinoic acid-RXRA	-1.798	-2.112	-0.489
PNPLA2	-1.794	-2.854	N/A
INSIG1	-1.777	2.388	N/A
TGFB3	-1.759	2.892	-0.307
CYP1B1	-1.755	N/A	-2.219
NFE2L2	-1.736	-4.51	N/A

EDN3	-1.664	2.377	-0.152
HNF4A	-1.662	-6.356	0.792
GATA1	-1.57	N/A	2.421
BMP4	-1.566	0.278	2.248
IGFBP2	-1.535	2.412	-0.758
SPARC	-1.513	-3.343	1.245
NFYA	-1.405	-2.561	N/A
RXRA	-1.358	-2.107	N/A
PRKCD	-1.313	1.35	2.113
TP53	-1.242	1.182	3.445
PPARD	-1.173	-2.878	N/A
N-cor	-1.108	2.219	N/A
RBL1	-1.048	-3.482	2.723
ERBB4	-1.033	-0.363	-2.781
PI3K (family)	-0.819	-0.901	-2.908
LPCAT3	-0.816	-2	N/A
YAP1	-0.812	3.7	-0.751
EGLN	-0.806	2.881	-2.798
FOXA1	-0.762	-2.047	-1.074
CCND1	-0.75	4.397	-4.599
NTRK2	-0.714	2.065	-1.474
Immunoglobulin	-0.705	0.585	-2.061
IRF2	-0.675	-2.095	1.682
ERK1/2	-0.673	3.051	0.112
VCAN	-0.647	-0.626	-3.448
E2F3	-0.62	3.687	-3.576
INSR	-0.572	-3.708	N/A
HNF1B	-0.57	-3.127	N/A
KDM1A	-0.561	2.179	-2.45
GLI1	-0.529	-1.601	-3.684
FN1	-0.468	2.51	N/A
RELA	-0.461	2.309	N/A
let-7	-0.441	-3.197	3.917
KLF5	-0.441	2.282	-1.76
ESR1	-0.402	3.03	-1.693
ERK	-0.401	3.305	N/A
VEGFA	-0.388	2.026	-2.222
THRB	-0.347	-2.043	N/A
mir-21	-0.332	-2.03	1.919
BTG2	-0.328	-2.54	0.238
ERBB2	-0.305	3.579	-6.116
NCOA2	-0.284	-2.07	N/A
VDR	-0.277	2.404	-1.55
Gcg	-0.258	-1.604	2.236
HGF	-0.213	3.723	-1.841
SNAI1	-0.191	1.424	-2.003
miR-155-5p (miRNAs w/seed UAAUGCU	-0.15	-2.587	1.53

IL2	-0.121	2.919	-2.15
BSG	-0.109	2.175	-0.579
FGF2	-0.088	2.133	1.077
miR-199a-5p (and other miRNAs w/seec	-0.057	-2.428	N/A
POR	0.07	2.686	0.724
FXR ligand-FXR-Retinoic acid-RXR?	0.098	-2.447	N/A
CD36	0.108	1.74	2.095
EZH2	0.122	2.147	N/A
S100A9	0.142	1.092	-3.309
IRF1	0.145	-2.302	-1.942
PRL	0.16	-1.264	-2.663
HDAC1	0.206	0.457	2.998
CDKN2A	0.215	-3.29	4.203
CSF2	0.216	3.361	-2.182
IRF3	0.241	-0.509	-2.659
FOXO4	0.269	-0.226	2.09
IFNB1	0.339	-2.403	-0.876
SMARCB1	0.346	-2.18	3.053
MGP	0.378	2.354	N/A
EFNA1	0.385	-2.034	N/A
SP110	0.392	-2.661	N/A
mir-8	0.413	-3.113	1.526
NCOR1	0.426	2.35	1.863
EHMT1	0.426	-1.301	2.111
RPTOR	0.445	-2.548	N/A
TAB1	0.454	2.216	0.945
PDLIM2	0.469	-0.871	-2.309
S100A8	0.478	1.37	-2.719
Vegf	0.491	3.789	-2.442
SYVN1	0.508	2.057	N/A
JUN	0.527	2.384	N/A
IFN Beta	0.533	-1.505	-2.248
SOX17	0.563	-2.155	N/A
RORA	0.567	0.279	-2.17
RAF1	0.622	3.092	-1.172
WT1	0.728	1.75	2.151
MAP3K7	0.816	2.117	-1.019
STAT1/3/5 dimer	0.816	2.236	N/A
FOSL1	0.827	4.16	N/A
TGFA	0.893	2.542	N/A
ESR2	0.9	2.452	-1.305
SLC27A2	0.91	3.707	N/A
SOX4	0.966	3.998	N/A
CCL5	1.017	0.388	-2.386
CEBPB	1.05	2.598	-4.085
SREBF1	1.093	-3.883	N/A
STAT3	1.176	0.004	2.516

PDGF BB	1.179	2.189	N/A
RABL6	1.192	2.683	-3.889
RUNX2	1.212	0.321	2.54
Mek	1.282	1.539	-2.036
DDX58	1.29	-2.795	-1.893
NGFR	1.359	-2.06	2.193
ELOVL3	1.387	2.478	N/A
SASH1	1.414	N/A	-2.333
HMOX1	1.426	-2.462	2.939
MAPK7	1.482	-3.193	N/A
MAP2K1/2	1.53	0.488	2.415
KLF6	1.615	2.33	N/A
MAVS	1.621	-2.675	-2.201
GSR	1.667	3.357	N/A
CEBPA	1.68	-2.782	1.303
TXNRD1	1.706	3.503	N/A
EGF	1.754	2.165	N/A
IL17A	1.763	2.199	N/A
NUPR1	1.889	-1.714	5.288
Nr1h	1.918	-2.765	N/A
MAP2K5	1.947	-3.095	-0.342
IL1A	1.997	2.816	N/A
TLR3	2.015	-0.156	N/A
NR3C1	2.066	-1.007	1.928
GPER1	2.074	-0.428	N/A
INPP5D	2.1	N/A	N/A
TNF	2.109	2.855	1.085
LUCAT1	2.121	-0.535	1.89
HFE	2.132	-2.219	N/A
SCAP	2.145	-4.227	N/A
CSF1	2.191	-1.064	N/A
GATA2	2.206	N/A	N/A
NCOA1	2.275	-1.303	N/A
TARDBP	2.36	-2.412	N/A
F3	2.421	2.67	-0.816
GCK	2.425	1.117	N/A
CSF	2.517	-0.218	N/A
IL1	2.541	2.743	0.645
SH3TC2	2.646	-2.673	N/A
BHLHE40	2.68	-0.2	0.104
SREBF2	2.745	-3.521	N/A
IL6	3.472	0.342	2.57
IL1B	3.553	1.373	0.888
E2f	N/A	4.41	-4.185
TBX2	N/A	4.168	-3.897
CDKN1A	N/A	-2.875	4.399
Rb	N/A	-3.455	3.395

RB1	N/A	-3.18	2.99
EP400	N/A	2.985	-3.162
MAP4K4	N/A	4.034	-2
IFNL1	N/A	-2.506	-3.242
BNIP3L	N/A	-2.412	2.72
E2F6	N/A	-1.897	3.051
FOXM1	N/A	3.493	-1.405
E2F2	N/A	2.644	-1.667
ERN1	N/A	-1.715	2.433
SIX2	N/A	-1.789	2.121
ACSS2	N/A	-3.827	N/A
EIF4G1	N/A	2.353	-1.342
DYRK1A	N/A	-2.279	1.353
NKX2-2-AS1	N/A	-3.606	N/A
PPARGC1B	N/A	-3.523	N/A
BACH1	N/A	3.498	N/A
MFSD2A	N/A	3.426	N/A
PTN	N/A	-3.418	N/A
TRIB3	N/A	1.039	2.177
ATP7B	N/A	-3.207	N/A
AIRE	N/A	-1	2
H2AZ1	N/A	0.956	-2
COMMD1	N/A	2.891	N/A
IGF2	N/A	-2.333	-0.555
CITED2	N/A	-2.011	0.842
XDH	N/A	2.793	N/A
ATF4	N/A	-2.747	N/A
let-7a-5p (and other miRNAs w/seed GA	N/A	-2.659	N/A
MIF	N/A	2.633	N/A
Irgm1	N/A	-0.316	2.274
ELAVL1	N/A	-0.36	-2.179
ZFP36	N/A	-2.483	N/A
IFNL4	N/A	N/A	-2.421
ETS1	N/A	2.363	N/A
C4BP	N/A	-2.236	N/A
TLR7	N/A	2.236	N/A
CHCHD5	N/A	-2.236	N/A
AGO2	N/A	2.233	N/A
TGIF2	N/A	2.224	N/A
SOD1	N/A	-2.2	N/A
CXCR4	N/A	2.113	N/A
Laminin (complex)	N/A	2.096	N/A
ALDH3A2	N/A	2	N/A
PPP2R2A	N/A	2	N/A

Table S7. Over-representation analysis of COP1 and HNF4A targeted hits. Analysis performed via WebGestalt, mapping shared FDR significant hits to enriched KEGG, GO and reactome gene sets. Only FDR significant hits are shown. Values are organized by decreasing enrichments ratios, to highlight relative biological contribution. Size, Gene Set Size; Expect: Expected value by chance (number of hits mapped to both input and geneset); Ratio: Enrichment ratio (number of actual hits mapped to input and geneset overlap vs number of hits predicted by chance). P value, nominal pvalue of overlap. FDR, False Discovery Rate.

GO (Biological_Process_noRedundant)						
Gene Set	Description	Size	Expect	Ratio	P Value	FDR
GO:0044282	small molecule catabolic process	401	5.40	4.45	8.8E-10	7.4E-07
GO:0015711	organic anion transport	431	5.80	3.79	8.1E-08	3.5E-05
GO:0055088	lipid homeostasis	116	1.56	7.05	4.5E-07	1.3E-04
GO:0010876	lipid localization	343	4.62	3.90	8.9E-07	1.9E-04
GO:0070482	response to oxygen levels	321	4.32	3.94	1.6E-06	2.7E-04
GO:0016042	lipid catabolic process	295	3.97	4.03	2.4E-06	3.4E-04
GO:0072376	protein activation cascade	90	1.21	7.43	3.3E-06	3.8E-04
GO:0002526	acute inflammatory response	143	1.92	5.72	3.6E-06	3.8E-04
GO:0001101	response to acid chemical	309	4.16	3.85	4.4E-06	4.1E-04
GO:0043627	response to estrogen	71	0.96	8.37	4.8E-06	4.1E-04
GO:0008202	steroid metabolic process	288	3.88	3.87	8.2E-06	6.3E-04
GO:0006631	fatty acid metabolic process	329	4.43	3.61	9.7E-06	6.8E-04
GO:0042180	cellular ketone metabolic process	170	2.29	4.81	1.9E-05	1.2E-03
GO:1901615	organic hydroxy compound metabolic process	474	6.38	2.98	2.2E-05	1.3E-03
GO:0043574	peroxisomal transport	66	0.89	7.88	2.8E-05	1.6E-03
GO:0019216	regulation of lipid metabolic process	360	4.84	3.30	2.9E-05	1.6E-03
GO:0006979	response to oxidative stress	406	5.46	3.11	3.5E-05	1.7E-03
GO:0051348	negative regulation of transferase activity	251	3.38	3.85	3.5E-05	1.7E-03
GO:0006732	coenzyme metabolic process	336	4.52	3.32	5.0E-05	2.2E-03
GO:0006520	cellular amino acid metabolic process	300	4.04	3.47	5.5E-05	2.3E-03
GO:0016053	organic acid biosynthetic process	380	5.11	3.13	5.6E-05	2.3E-03
GO:0015850	organic hydroxy compound transport	227	3.05	3.93	5.8E-05	2.3E-03
GO:0043062	extracellular structure organization	385	5.18	3.09	6.5E-05	2.3E-03
GO:0009636	response to toxic substance	470	6.33	2.85	6.6E-05	2.3E-03

GO:0007031	peroxisome organization	77	1.04	6.76	7.8E-05	2.6E-03
GO:0097006	regulation of plasma lipoprotein particle levels	78	1.05	6.67	8.4E-05	2.8E-03
GO:0006790	sulfur compound metabolic process	333	4.48	3.12	1.7E-04	5.3E-03
GO:0015849	organic acid transport	296	3.98	3.26	1.9E-04	5.7E-03
GO:0010038	response to metal ion	339	4.56	3.07	2.0E-04	5.9E-03
GO:0046677	response to antibiotic	299	4.02	3.23	2.1E-04	5.9E-03
GO:0051604	protein maturation	307	4.13	3.15	2.7E-04	7.3E-03
GO:0002791	regulation of peptide secretion	439	5.91	2.71	3.0E-04	7.8E-03
GO:0071825	protein-lipid complex subunit organization	45	0.61	8.26	3.3E-04	8.5E-03
GO:0061008	hepaticobiliary system development	133	1.79	4.47	4.3E-04	1.1E-02
GO:0062012	regulation of small molecule metabolic process	324	4.36	2.98	4.5E-04	1.1E-02
GO:0048732	gland development	414	5.57	2.69	4.9E-04	1.1E-02
GO:0071706	tumor necrosis factor superfamily cytokine produc	136	1.83	4.37	5.0E-04	1.1E-02
GO:0034367	protein-containing complex remodeling	28	0.38	10.62	5.1E-04	1.1E-02
GO:0009743	response to carbohydrate	213	2.87	3.49	6.2E-04	1.4E-02
GO:0005996	monosaccharide metabolic process	257	3.46	3.18	7.3E-04	1.5E-02
GO:0016052	carbohydrate catabolic process	150	2.02	3.96	9.6E-04	1.8E-02
GO:0033500	carbohydrate homeostasis	222	2.99	3.01	3.1E-03	4.5E-02
GO:0061458	reproductive system development	406	5.46	2.38	3.4E-03	4.7E-02
GO:0006979	response to oxidative stress	406	5.46	3.11	3.5E-05	1.7E-03
GO:0043062	extracellular structure organization	385	5.18	3.09	6.5E-05	2.3E-03
GO:0010038	response to metal ion	339	4.56	3.07	2.0E-04	5.9E-03
GO:0033500	carbohydrate homeostasis	222	2.99	3.01	3.1E-03	4.5E-02
GO:0030258	lipid modification	274	3.69	2.98	1.2E-03	2.1E-02
GO:0062012	regulation of small molecule metabolic process	324	4.36	2.98	4.5E-04	1.1E-02
GO:1901615	organic hydroxy compound metabolic process	474	6.38	2.98	2.2E-05	1.3E-03
GO:0009914	hormone transport	308	4.15	2.90	9.6E-04	1.8E-02
GO:0009636	response to toxic substance	470	6.33	2.85	6.6E-05	2.3E-03
GO:0051188	cofactor biosynthetic process	264	3.55	2.81	3.1E-03	4.5E-02
GO:0050727	regulation of inflammatory response	348	4.68	2.78	8.7E-04	1.8E-02
GO:0002791	regulation of peptide secretion	439	5.91	2.71	3.0E-04	7.8E-03
GO:0048732	gland development	414	5.57	2.69	4.9E-04	1.1E-02
GO:0048545	response to steroid hormone	364	4.90	2.65	1.3E-03	2.2E-02
GO:0042326	negative regulation of phosphorylation	395	5.32	2.63	9.3E-04	1.8E-02

GO:0052547	regulation of peptidase activity	398	5.36	2.61	1.0E-03	1.8E-02
GO:0051047	positive regulation of secretion	388	5.22	2.49	2.3E-03	3.5E-02
GO:0050878	regulation of body fluid levels	458	6.16	2.43	1.4E-03	2.3E-02
GO:0061458	reproductive system development	406	5.46	2.38	3.4E-03	4.7E-02

REACTOME

Gene Set	Description	Size	Expect	Ratio	P Value	FDR
R-HSA-1430728	Metabolism	1957	30.49	2.00	1.0E-08	1.7E-05
R-HSA-192105	Synthesis of bile acids and bile salts	32	0.50	14.04	4.7E-07	4.0E-04
R-HSA-193368	Synthesis of bile acids and bile salts via 7alpha-hydrox	22	0.34	17.50	7.9E-07	4.5E-04
R-HSA-194068	Bile acid and bile salt metabolism	41	0.64	10.96	2.8E-06	1.2E-03
R-HSA-977606	Regulation of Complement cascade	45	0.70	9.98	5.4E-06	1.8E-03
R-HSA-166658	Complement cascade	56	0.87	8.02	2.4E-05	5.9E-03
R-HSA-211945	Phase I - Functionalization of compounds	100	1.56	5.78	2.4E-05	5.9E-03
R-HSA-9033241	Peroxisomal protein import	60	0.93	7.49	3.8E-05	8.0E-03
R-HSA-1474228	Degradation of the extracellular matrix	136	2.12	4.72	5.0E-05	9.5E-03
R-HSA-174577	Activation of C3 and C5	8	0.12	24.07	2.0E-04	3.0E-02
R-HSA-166665	Terminal pathway of complement	8	0.12	24.07	2.0E-04	3.0E-02
R-HSA-211859	Biological oxidations	195	3.04	3.62	2.3E-04	3.2E-02
R-HSA-8957275	Post-translational protein phosphorylation	106	1.65	4.84	2.4E-04	3.2E-02

KEGG

Gene Set	Description	Size	Expect	Ratio	P Value	FDR
hsa04610	Complement and coagulation cascades	79	1.41	7.10	1.1E-06	3.7E-04
hsa00120	Primary bile acid biosynthesis	16	0.29	17.53	6.2E-06	1.0E-03
hsa03320	PPAR signaling pathway	67	1.19	5.86	1.7E-04	1.9E-02
hsa04146	Peroxisome	80	1.43	4.91	5.2E-04	3.5E-02
hsa04726	Serotonergic synapse	105	1.87	4.27	5.3E-04	3.5E-02
hsa04950	Maturity onset diabetes of the young	25	0.45	8.98	9.1E-04	4.9E-02

Table S8. Comparison of Gene list from Lee et al (Suppl Table 2) with values from current study. 78 Gene list from Lee et al, describing genes impacted similarly in HuH-7 and HepG2 was converted to a 74 gene list via the Transcriptome Analysis Console. Values from Lee et al are available at the Gene Expression Omnibus dataset GSE21955. Upregulated and downregulated genes in response to COP1 suppression are highlighted in red and green respectively.

**HepG2 (current)**			**HepG2 (Lee et al)**		
Gene Symbol	Fold Chan	pValue	Gene Symbol	Fold Change (Log2)	pValue
AARS	-1.07	0.5923	AARS	-1.024	0
ABL1	1	0.3505	ABL1	-1.307	0.0004
ALDH18A1	-1.02	0.9913	ALDH18A1	-1.268	0.0006
ARIH2	-1.05	0.9615	ARIH2	-1.064	0.0018
ARIH2	-1.08	0.3728			
ARIH2	-1.01	0.4357			
B4GALT5	1.59	0.0006	B4GALT5	-1.214	0.0014
BOLA3	-1.13	0.098	BOLA3	-1.746	0
C6orf106	-1.3	0.0247	C6orf106	-1.287	0
C17orf58	-1.08	0.2769	C17orf58	-1.515	0.0002
CANX	-1.03	0.8011	CANX	-3.516	0.0006
CCNI	1.13	0.2479	CCNI	-1.688	0
CETN2	1.04	0.736	CETN2	-1.173	0
COMMD1	-1.22	0.1158	COMMD1	-1.315	0
COQ9	-1.04	0.5042	COQ9	-1.49	0.0004
COX7A2	-1.15	0.1134	COX7A2	-2.216	0.0016
CPD	-1.17	0.025	CPD	-1.602	0.0012
CTDSPL	1.45	0.0016	CTDSPL	-1.088	0.0004
CTRC	1.05	0.4831	CTRC	1.286	0.0037
EPB41L4B	-1.11	0.2702	EPB41L4B	-1.091	0
ETV4	-1.4	0.002	ETV4	-1.127	0
FADD	-1.01	0.4299	FADD	-1.836	0.0016
FAM168B	1.16	0.0884	FAM168B	-1.086	0
FBXW4	-1	0.7858	FBXW4	-1.085	0
FHL2	1.32	0.0024	FHL2	1.036	0.0004

FOXJ3	1.21	0.1518	FOXJ3	-1.707	0.0002
FOXO3	-1.02	0.3337	FOXO3	-1.109	0.0048
FZD9	1.16	0.4699	FZD9	-1.455	0.0002
GLIPR1	2.92	8.13E-07	GLIPR1	1.86	0.0006
GLUD1	-1.23	0.0257	GLUD1	-1.716	0.0037
HIST1H2AC	-1.12	0.1045	HIST1H2AC	1.192	0
IDH1	-1.5	0.0003	IDH1	-1.515	0
LEPR; LEPROT	1.02	0.8324	LEPROT	-1.82	0
LETM1	-1.16	0.1599	LETM1	-1.942	0
MDP1; NEDD8-MDF	1	0.9445	NEDD8	-1.108	0
MORC2	1.09	0.5599	MORC2	-1.324	0
MRPL18	1.09	0.2511	MRPL18	-2.069	0
NETO2	1.68	0.0003	NETO2	-3.022	0.0006
NFIX	-1.05	0.7755	NFIX	-1.425	0
NQO1	-1.71	4.02E-05	NQO1	-2.131	0
NUDT2	-1.01	0.4154	NUDT2	-1.186	0
PLEKHB2	1	0.888	PLEKHB2	1.044	0
POLR2C	-1.01	0.3243	POLR2C	-1.612	0.0029
RCC2	1.17	0.035	RCC2	-2.127	0
RFWD2	-2.71	1.52E-05	RFWD2	-2.034	0
RPAIN	1.06	0.8175	RPAIN	-1.116	0.0006
RPUSD3	1.02	0.9947	RPUSD3	-1.192	0.0002
SCMH1	-1.03	0.6343	SCMH1	-1.061	0.0018
SDC1	1.62	0.0018	SDC1	-1.255	0.0004
SDC4	1.5	0.0033	SDC4	1.044	0
SLC31A2	1.04	0.6018	SLC31A2	1.047	0.0006
SOCS2	1.12	0.2457	SOCS2	1.195	0
SPOCK2	-1.39	0.0145	SPOCK2	-2.314	0.0002
ST6GALNAC6	-1.21	0.0412	ST6GALNAC6	-1.19	0
SURF4	-1.05	0.2975	SURF4	-1.914	0
TALDO1	-1.34	0.013	TALDO1	-1.642	0.0018
TAP1	-1.14	0.2619	TAP1	1.054	0
TFPI	1.09	0.2803	TFPI	-1.798	0.0018
THAP11	1.04	0.9195	THAP11	-1.541	0

TIMM23	-1.07	0.3857	TIMM23	-1.639	0.0008
TIMM23B; AGAP6;	-1.29	0.0346			
TIMP2; CEP295NL	1.35	0.0047	TIMP2	1.107	0
TMCO3	-1.3	0.0462	TMCO3	-1.24	0.004
TMEM30A; COX7A	1.13	0.0875			
TNFRSF21	-1.18	0.0422	TNFRSF21	-1.871	0
TRIM35	1	0.8196	TRIM35	1.117	0
TUFT1	-1.27	0.0157	TUFT1	1.004	0
UBE2G2	-1.08	0.3353	UBE2G2	-2.013	0.0012
UBE2Q1	1.04	0.4841	UBE2Q1	-1.897	0
UBE2Z	1.15	0.1203	UBE2Z	-1.279	0.0008
UBFD1	-1.1	0.1842	UBFD1	-1.081	0.0004
UBIAD1	1.07	0.2348	UBIAD1	-1.617	0
ZHX3	1.01	0.3078	ZHX3	-1.072	0.0012
ZNF358	1.01	0.2765	ZNF358	-1.14	0.0018

**HuH7 (current)**			**Huh7 (Lee et al)**		
Gene Symbol	Fold Chang	pValue		Fold Change (Log2)	pValue
AARS	1.04	0.8703	AARS	-1.061	0
ABL1	-1.16	0.2362	ABL1	-1.006	0
ALDH18A1	1.3	0.0277	ALDH18A1	-1.526	0.0007
ARIH2	-1.23	0.05	ARIH2	-1.112	0
ARIH2	1.01	0.9433			
ARIH2	1.09	0.4479			
B4GALT5	1.14	0.8097	B4GALT5	-1.115	0.0086
BOLA3	-1.26	0.0952	BOLA3	-1.991	0.0092
C6orf106	1.14	0.4155	C6orf106	-1.423	0.0078
C17orf58	-1.1	0.6015	C17orf58	-1.207	0
CANX	1.02	0.871	CANX	-3.083	0
CCNI	-1.06	0.5705	CCNI	-1.819	0
CETN2	-1.05	0.5515	CETN2	-1.16	0.0006
COMMD1	-1.21	0.1006	COMMD1	-2.195	0.0028
COQ9	1.06	0.5114	COQ9	-2.017	0

COX7A2	-1.07	0.6507	COX7A2	-2.826	0
CPD	1.12	0.3535	CPD	-1.321	0.005
CTDSPL	1.17	0.2574	CTDSPL	-2.911	0
CTRC	-1.17	0.2777	CTRC	-1.373	0.001
EPB41L4B	-1.05	0.9708	EPB41L4B	-2.199	0
ETV4	-1.52	0.0081	ETV4	-3.142	0
FADD	1.07	0.9915	FADD	-1.684	0.0061
FAM168B	1.42	0.04	FAM168B	-1.118	0
FBXW4	-1.13	0.8449	FBXW4	-1.03	0.0035
FHL2	-1.25	0.1981	FHL2	1.138	0
FOXJ3	-1.04	0.3585	FOXJ3	-1.335	0
FOXO3	-1	0.9975	FOXO3	-1.372	0.0082
FZD9	1.16	0.4633	FZD9	-3.333	0
GLIPR1	-1.47	0.2894	GLIPR1	3.002	0.0003
GLUD1	1.09	0.4917	GLUD1	-1.5	0.0097
HIST1H2AC	1.27	0.0658	HIST1H2AC	1.366	0.0089
IDH1	1	0.9332	IDH1	-1.017	0.008
LEPR; LEPROT	1.1	0.8202	LEPROT	-1.98	0.0014
LETM1	-1.17	0.4937	LETM1	-1.72	0.0003
MDP1; NEDD8-MDP1;	-1.17	0.335	NEDD8	-1.146	0.0062
MORC2	1.22	0.4537	MORC2	-1.156	0.009
MRPL18	-1.1	0.1999	MRPL18	-2.199	0.0049
NETO2	1.12	0.8749	NETO2	-1.392	0.0025
NFIX	-1.18	0.1033	NFIX	-2.388	0
NQO1	-1.14	0.5158	NQO1	-2.418	0
NUDT2	1.2	0.0478	NUDT2	-1.64	0
PLEKHB2	-1.14	0.0672	PLEKHB2	1.278	0.0066
POLR2C	1.08	0.8576	POLR2C	-1.091	0
RCC2	1.33	0.1616	RCC2	-1.419	0.0019
RFWD2	-3.35	0.001	RFWD2	-2.695	0
RPAIN	1.13	0.2309	RPAIN	-1.602	0.0015
RPUSD3	1.1	0.6669	RPUSD3	-1.501	0.0025
SCMH1	-1.08	0.4546	SCMH1	-1.029	0.0068
SDC1	1.15	0.5305	SDC1	-2.118	0

SDC4	1.29	0.5247	SDC4	1.189	0.0059
SLC31A2	-1.12	0.2158	SLC31A2	1.794	0
SOCS2	1.17	0.5212	SOCS2	2.182	0.0008
SPOCK2	1.07	0.5896	SPOCK2	-2.23	0
ST6GALNAC6	1.25	0.0905	ST6GALNAC6	-1.538	0.0036
SURF4	1.2	0.1395	SURF4	-1.886	0
TALDO1	-1.18	0.0887	TALDO1	-1.491	0.0023
TAP1	-1.14	0.6264	TAP1	2	0
TFPI	1.24	0.3774	TFPI	-1.28	0.0085
THAP11	-1.13	0.6441	THAP11	-1.442	0
TIMM23	-1.11	0.394	TIMM23	-1.399	0.0072
TIMM23B; AGAP6; TIN	1.17	0.3508			
TIMP2; CEP295NL	1.03	0.5754	TIMP2	1.103	0.0004
TMCO3	1.02	0.8982	TMCO3	-1.705	0
TMEM30A; COX7A2	1	0.8953			
TNFRSF21	1.16	0.4267	TNFRSF21	-1.276	0
TRIM35	1.06	0.5854	TRIM35	1.074	0.0028
TUFT1	1.02	0.8536	TUFT1	1.868	0
UBE2G2	-1.09	0.2776	UBE2G2	-1.727	0.002
UBE2Q1	-1.09	0.3168	UBE2Q1	-1.702	0
UBE2Z	1.08	0.4197	UBE2Z	-1.468	0.0032
UBFD1	1.2	0.2846	UBFD1	-1.449	0.0021
UBIAD1	1.12	0.4943	UBIAD1	-2.501	0.0002
ZHX3	-1.04	0.6264	ZHX3	-1.383	0
ZNF358	1	0.8263	ZNF358	-2.328	0.0019
